# Reiterative Enrichment and Authentication of CRISPRi Targets (REACT) identifies the proteasome as a key contributor to HIV-1 latency

**DOI:** 10.1371/journal.ppat.1007498

**Published:** 2019-01-15

**Authors:** Zichong Li, Jun Wu, Leonard Chavez, Rebecca Hoh, Steven G. Deeks, Satish K. Pillai, Qiang Zhou

**Affiliations:** 1 Department of Molecular and Cell Biology, University of California, Berkeley, California, United States of America; 2 School of Pharmaceutical Sciences, Xiamen University, Xiamen, China; 3 Vitalant Research Institute, San Francisco, California, United States of America; 4 University of California, San Francisco, California, United States of America; Miller School of Medicine, UNITED STATES

## Abstract

The establishment of HIV-1 latency gives rise to persistent chronic infection that requires life-long treatment. To reverse latency for viral eradiation, the HIV-1 Tat protein and its associated ELL2-containing Super Elongation Complexes (ELL2-SECs) are essential to activate HIV-1 transcription. Despite efforts to identify effective latency-reversing agents (LRA), avenues for exposing latent HIV-1 remain inadequate, prompting the need to identify novel LRA targets. Here, by conducting a CRISPR interference-based screen to reiteratively enrich loss-of-function genotypes that increase HIV-1 transcription in latently infected CD4^+^ T cells, we have discovered a key role of the proteasome in maintaining viral latency. Downregulating or inhibiting the proteasome promotes Tat-transactivation in cell line models. Furthermore, the FDA-approved proteasome inhibitors bortezomib and carfilzomib strongly synergize with existing LRAs to reactivate HIV-1 in CD4^+^ T cells from antiretroviral therapy-suppressed individuals without inducing cell activation or proliferation. Mechanistically, downregulating/inhibiting the proteasome elevates the levels of ELL2 and ELL2-SECs to enable Tat-transactivation, indicating the proteasome-ELL2 axis as a key regulator of HIV-1 latency and promising target for therapeutic intervention.

## Author summary

To cure chronic HIV-1 infection requires reversal of HIV-1 latency from latently infected CD4^+^ T cells. A key step in HIV latency reversal is the recruitment of Super Elongation Complexes (SECs) that contain ELL2 by an HIV-encoded protein, Tat, to activate proviral transcription. To identify novel drug targets, we conducted a CRISPRi-based screen to enrich the sgRNAs that increase HIV transcription in latently infected CD4^+^ T cells. Three of the six most prominent hits in our screen are proteasome subunits. We further proved that antagonizing the proteasome promotes Tat-induced HIV-1 transcription in cell line-based latency models. Furthermore, we found that two FDA-approved proteasome inhibitors strongly synergize with existing LRAs *ex vivo* without inducing cell activation or proliferation. We further found that antagonizing the proteasome elevates the levels of ELL2 and ELL2-containing SECs in the cells, thus enabling Tat-transactivation. These results indicate that the proteasome-ELL2 axis is a key regulator of HIV-1 latency could potentially be targeted for therapeutic interventions.

## Introduction

Transcriptional silence of integrated HIV-1 proviruses in a minority of infected CD4^+^ T cells is a key signature of the latent viral reservoirs that necessitate a lifelong antiretroviral therapy (ART) to maintain their silence [1,2]. Strategies to expose the latently infected cells for immune recognition and clearance in individuals on ART rely on latency reversing agents (LRAs) to reactivate proviral transcription [3,4,5]. To date, multiple clinical trials have tested a variety of LRAs that are dominated by histone deacetylase (HDAC) inhibitors and NF-κB agonists [6]. However, only modest increases in viral transcription with little to no reservoir reduction are induced by these drugs [7].

Compared with the mechanisms used by the HDAC inhibitors and NF-κB agonists to relax chromatin and recruit RNA polymerase (Pol) II to the HIV-1 promoter, respectively, a less leveraged but arguably more specific and prominent feature of the HIV-1 transcriptional control is the Tat-dependent transition of Pol II from promoter-proximal pausing to productive elongation [8,9]. This rate-limiting step fuels a potent positive-feedback circuit to activate viral transcription without causing T cell activation [10].

Mechanistically, Tat stimulates HIV-1 transcriptional elongation by recruiting a specific host co-activator, the human Super Elongation Complex (SEC) [11,12], to the paused Pol II through forming the Tat-SEC complex on the TAR RNA, a stem-loop structure located at the 5’ end of the nascent viral transcript [13,14]. The two critical components of the SEC, P-TEFb and ELL2, stimulate Pol II elongation by different mechanisms and can thus synergistically induce the production of full-length viral transcripts [8]. In addition to residing in the SEC, P-TEFb also interacts with the bromodomain protein Brd4, which competitively inhibits the Tat-SEC interaction [15,16]. The small molecule suppressor JQ1 binds to Brd4 to antagonize its inhibitory action on Tat-SEC, leading to the activation of HIV-1 transcription and latency reversal [17,18,19,20]. Notably, JQ1 is shown to synergize with other LRAs to reactivate latent HIV-1 in a number of *ex vivo* experiments involving CD4^+^ T cells from the ART-suppressed individuals [21,22,23,24].

A typical SEC contains P-TEFb, as well as one of each of the three pairs of homologous proteins: ELL1/ELL2, AFF1/AFF4, and ENL/AF9. Owing to the ability of these proteins to create multiple different combinations among them, a family of related SEC complexes exists in cells. Our recent study shows that a low-abundance subset of SECs containing ELL2 and AFF1 play a predominant role in cooperating with Tat to reverse HIV-1 latency [25]. In fact, by simply increasing the cellular level of ELL2, a highly unstable protein due to its polyubiquitination by the E3 ubiquitin ligase Siah1 and subsequent degradation by the proteasome [11,26,27], it was possible to activate latent HIV-1 without using any drugs [25].

The complexity of the mechanisms that contribute to HIV-1 latency suggests that combinatorial LRAs from distinct mechanistic classes are necessary to expose the hidden viruses [3]. To identify novel classes of LRAs that cooperate well with the existing ones, we developed a genetic screen based on CRISPR interference (CRISPRi) [28] to look for additional host restriction factors that may represent previously unrecognized drug targets. By selecting authentic and effective CRISPRi targets through reiterative enrichments, we have identified several subunits of the proteasome as novel host factors that strongly inhibit HIV-1 transcription and promote latency. Our data indicate that the proteasome preferentially inhibits the Tat-dependent HIV-1 transcription by decreasing the cellular level of ELL2, which in turn prevents formation of the ELL2-containing SECs. Furthermore, several FDA-approved proteasome inhibitors are shown to act synergistically with the existing LRAs to activate HIV-1 without inducing cell activation or proliferation in both cell line-based latency models and primary T cells from HIV-1-infected and ART-suppressed individuals. Collectively, our data indicate that targeting the proteasome-ELL2 axis provides a new avenue to expose the latent HIV-1 proviruses.

## Results

### Reiterative Enrichment and Authentication of CRISPRi Targets (REACT) identifies novel HIV-1 restriction factors in Jurkat 2D10 cells

**Fig 1:**
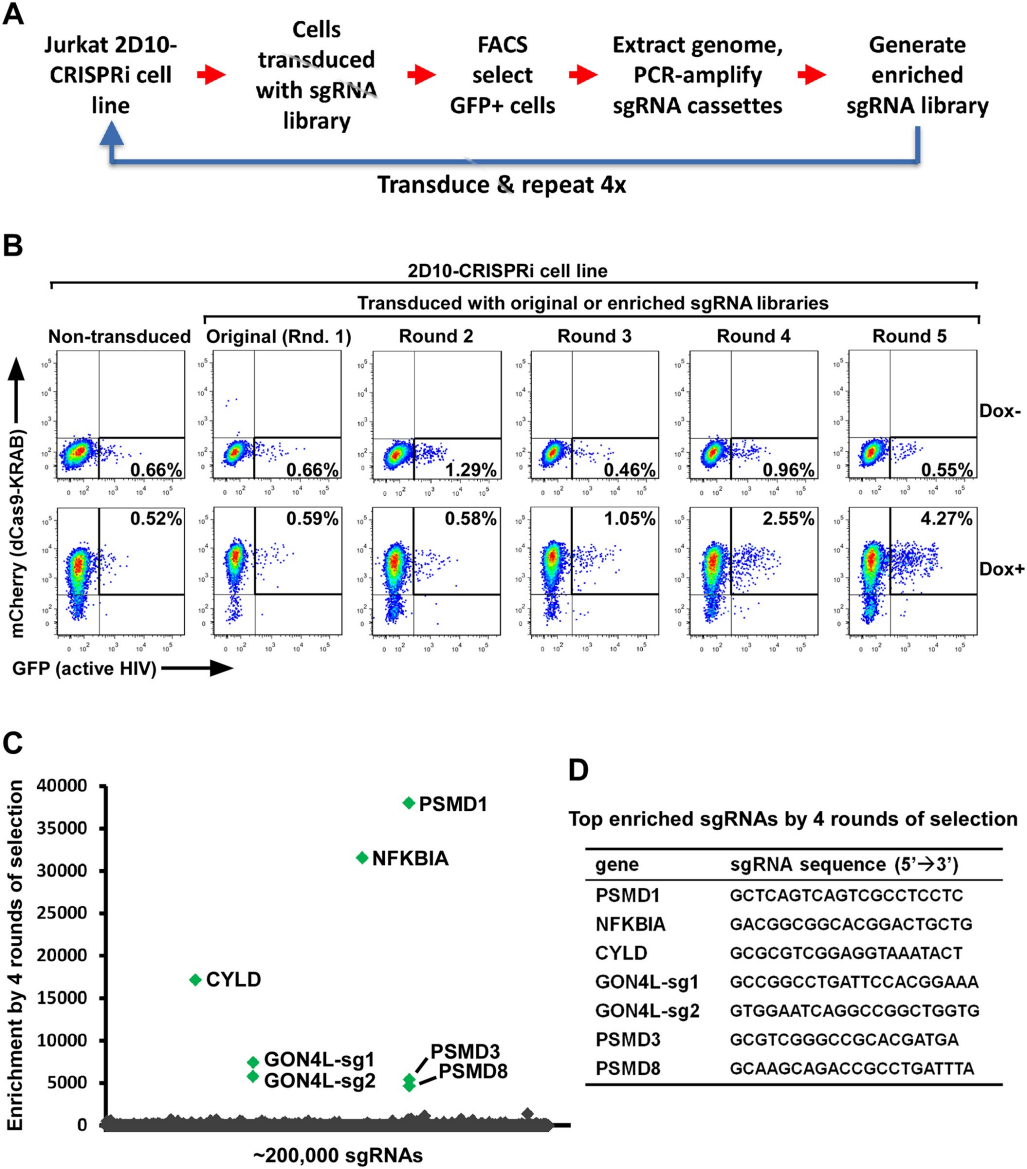
Reiterative Enrichment and Authentication of CRISPRi Targets (REACT) identifies novel HIV-1 restriction factors in Jurkat 2D10 cells. **A**. A diagram showing the procedure of REACT that involves repeated cycles of CRISPRi, FACS-selection of GFP+ cells, and subcloning of the sgRNA library to enrich the sgRNA sequences that target host HIV-1 restriction factors. The 2D10-based TetOn mCherry-dCas9-KRAB cell line (called 2D10-CRISPRi) was used. **B**. Representative FACS plots of 2D10-CRISPRi cells non-transduced or transduced by original or enriched sgRNA libraries. The cells were first treated with either DMSO (Dox-) or doxycycline (Dox+) to induce the expression of the KRAB-dCas9-HA-P2A-mCherry fusion protein and then selected by FACS for the GFP+ cells harboring activated HIV-1. **C**. The round 4-enriched and original sgRNA libraries were subjected to high throughput sequencing and the fold of enrichment for each sgRNA was calculated based on its reads per million in the round 4-enriched library divided by those in the original library and presented on a scatter plot, with the 7 most significantly enriched sequences highlighted in green. **D**. Shown are the sequences of the 7 sgRNA highlighted in C and their target genes.

To identify novel human genes that inhibit HIV-1 expression, we set up a screen for the loss-of-function genotypes that could lead to the activation of latent HIV-1 provirus in the Jurkat-based 2D10 cell line, a widely used post-integration latency model with the d2EGFP-coding sequence in place of the viral *nef* gene in the proviral genome [29]. We first generated a 2D10-based TetOn mCherry-dCas9-KRAB cell line (named 2D10-CRISPRi) by adapting an inducible CRISPRi platform [30]. The loss-of-function genotypes were produced in this cell line by stably transducing a whole genome sgRNA library containing a total of ~200,000 sgRNAs at an average of 10 per gene [30]. Three days after the doxycycline (Dox)-induced production of the dCas9-KRAB fusion, the cells were subjected to fluorescence-activated cell sorting (FACS) to isolate the GFP+ cells containing activated HIV-1 (Fig 1A).

Because the first round of selection did not yield any positive signals that were above the background (Fig 1B), we decided to repeat the procedure a few more times in the hope of enriching the desired genotypes (Fig 1A). To do this, the sgRNA sequences were PCR-amplified from the genome of GFP+ cells isolated from the previous round of FACS and cloned into the empty vector to generate an enriched sgRNA library, which was then transduced into the original 2D10-CRISPRi cells for the next round of selection (Fig 1A). This procedure, called the Reiterative Enrichment and Authentication of CRISPRi Targets or REACT, was repeated 4 times.

As expected, the CRISPRi-induced HIV-1 activation began to noticeably increase in the cell population starting from round 3 (1.06% GFP+ cells under Dox+ vs. 0.46% spontaneous reactivation under Dox- conditions) and culminating in round 5 of REACT (4.27% vs. 0.55%; Fig 1B). High-throughput sequencings of the sgRNA libraries enriched by rounds 1, 2, 3, and 4 demonstrate a progressive enrichment of several main hits from background noise (Fig 1C and S1 Fig). The 7 most significantly enriched sgRNAs target 6 different genes: PSMD1, NFKBIA, CYLD, GON4L, PSMD3, and PSMD8 (Fig 1D). Among these, NFKBIA (aka. IκBα, an inhibitor of NF-κB) and CYLD (a deubiquitinase for NFKBIA) have been reported to encode suppressors of HIV-1 transcription [31,32]. It is thus unsurprising that their silencing caused viral activation. On the other hand, PSMD1, PSMD3, and PSMD8, all encoding the canonical proteasome subunits, and GON4L, which encodes a protein found in a transcriptional co-repressor complex with HDAC1 [33], represent previously unreported and potentially novel host restriction factors for HIV-1.

### Proteasomal subunits identified by REACT inhibit Tat-dependent HIV-1 transcription

**Fig 2:**
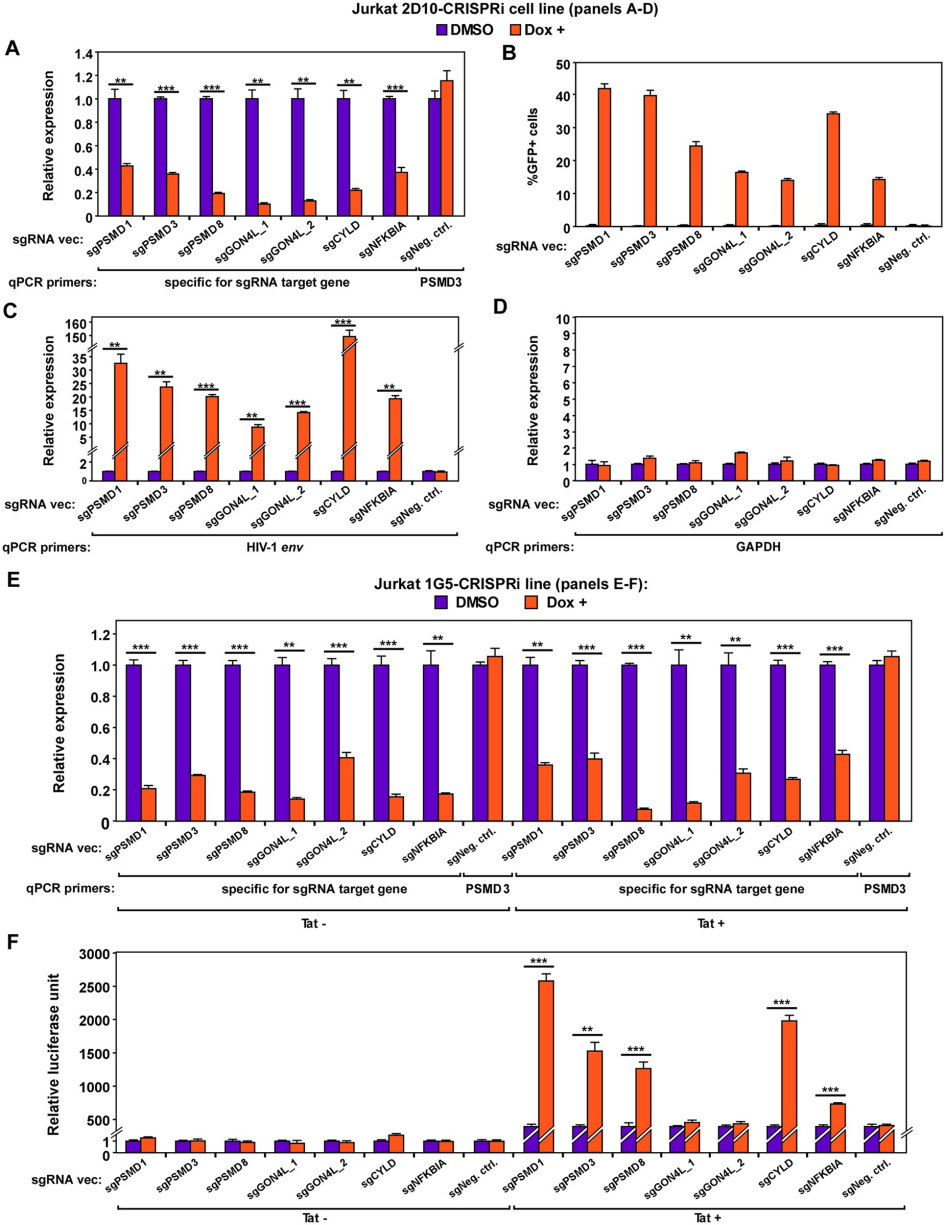
Verification of target specificity and HIV-1 activation potential of the 7 sgRNAs identified by REACT in 2D10 and 1G5-/+Tat cells. **A., C., D**., **& E**. The Jurkat-based 2D10-CRISPRi cells (A, C & D) or Jurkat-based 1G5-CRISPRi and 1G5+Tat-CRISPRi cells (E) were stably transduced with the indicated sgRNA expression vectors and then treated with DMSO or Dox. Aliquots of the cells were subjected to RT-qPCR analysis of the mRNA levels of the genes that are denoted by their corresponding qPCR primers. For each gene, the mRNA level detected in the DMSO-treated cells was set to 1. **B**. Results of FACS analysis of the percentages of GFP+ cells within the various cell populations obtained in A. **F**. Results of luciferase activities measured in extracts of the cells described in E. In all panels, error bars represent mean +/- standard deviation (SD) from three experimental replicates. In A, C, E, & F, asterisks denote levels of statistical significance calculated by two-tailed Student’s *t*-test (*: p<0.05, **: p<0.01, and ***: p<0.001).

To verify that the 6 genes identified by REACT indeed encode the restriction factors that promote HIV-1 latency, we synthesized and cloned the top 7 sgRNA hits and a negative control sequence into the empty vector used to generate the library, and stably transduced them into the 2D10-CRISPRi cells. The RT-qPCR and FACS analyses indicate that the 7 sgRNAs but not the negative control downregulated the expression of their respective target genes (Fig 2A) and efficiently activated HIV-1 (Fig 2B). Further RT-qPCR analyses demonstrated that after the 6 genes were downregulated by CRISPRi, the HIV-1 *env* mRNA level increased by one to two orders of magnitude (Fig 2C), whereas the cellular GAPDH transcript remained mostly unchanged (Fig 2D).

To further confirm that the proteasomal subunits can be downregulated to activate latent HIV-1, we used siRNAs to knock down the expression of PSMD1, PSMD3, as well as a non-proteasomal REACT target CYLD in two different HIV-1 latency model cell lines, Jurkat 2D10 [29] and J-Lat A2, which contains an integrated, transcriptionally silent LTR-Tat-Flag-IRES-EGFP cassette [34]. Like the CRISPRi-induced silencing, the knockdown (KD) by RNA interference (RNAi) significantly reactivated latent HIV-1 and enhanced mRNA production from the HIV-1 LTR but not the GAPDH promoter in both systems (S2A–S2F Fig).

Transcriptional silencing leads to HIV-1 latency. It is thus important to determine whether the 6 genes identified by REACT directly affect HIV-1 transcription. Specifically, we asked whether they influence Tat-transactivation, which is the most prominent feature of HIV-1 transcription. To this end, we examined the impact of the CRISPRi-induced downregulation of the 6 genes on expression of an integrated, HIV-1 LTR-driven luciferase reporter gene in Jurkat-based 1G5 [35] and 1G5+Tat cell lines [36]. The data indicate that downregulating PSMD1, PSMD3, PSMD8, NFKBIA and CYLD significantly increased the LTR-driven luciferase expression only in 1G5+Tat cells that constitutively express Tat (Fig 2E & 2F). In contrast, targeting GON4L by two different sgRNAs did not increase the LTR activity in either cell line. Together, these data implicate the proteasomal subunits PSMD1, PSMD3, and PSMD8 as novel host factors that inhibit Tat-dependent HIV-1 transcription and promote viral latency.

### Downregulating proteasomal core subunits or inhibiting proteasomal activity promotes HIV-1 transcription and latency reversal in cell line models

**Fig 3:**
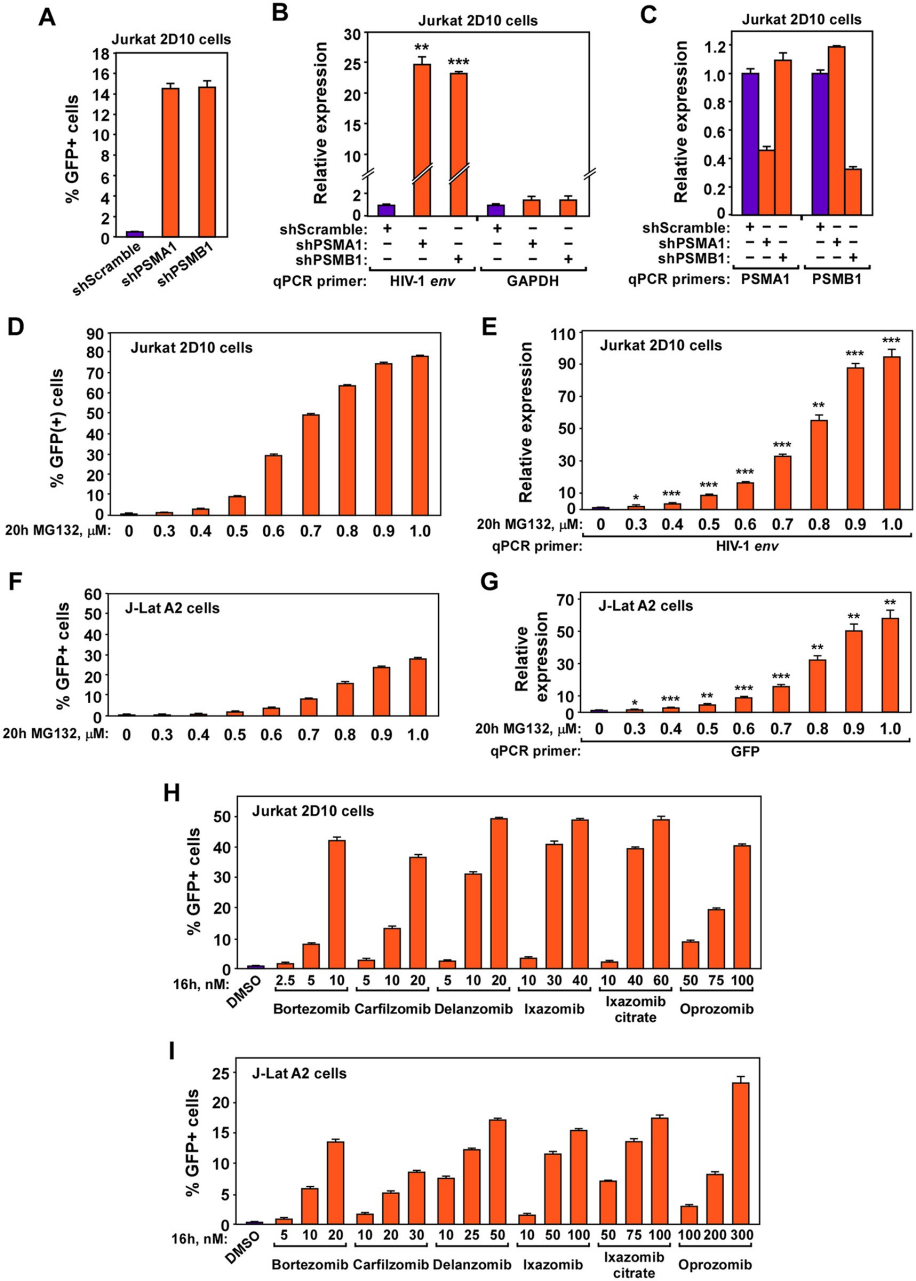
Downregulating proteasomal core subunits or inhibiting proteasomal activity promotes HIV-1 transcription and latency reversal in cell line models. **A., B., & C**. Jurkat 2D10 cells were nucleofected with the indicated siRNAs. Aliquots of the cells were subjected to FACS analysis to detect the percentages of GFP+ cells in each population (A) or RT-qPCR analysis of the mRNA levels produced by the genes labeled at the bottom (B & C). **D., E., F., G., H., & I**. Jurkat 2D10 (D, E, & H) or J-Lat A2 cells (F, G, & I) were treated with the indicated proteasome inhibitors at the indicated concentrations for either 16 or 20 hours as labeled. The treated cells were then subjected to FACS analysis to determine the percentages of GFP+ cells in each population (D, F, H, & I) or RT-qPCR analysis to examine the mRNA levels from the genes denoted by their specific qPCR primer (E & G). In all panels, error bars represent mean +/- SD from three experimental replicates. In B, E, & G, asterisks denote levels of statistical significance calculated by two-tailed Student’s *t*-test (*: p<0.05, **: p<0.01, and ***: p<0.001).

Since PSMD1, PSMD3 and PSMD8 are all located in the 19S regulatory particle of the 26S proteasome [37], we asked whether subunits in the 20S core particle also restrict HIV-1 activation. To answer this question, we used shRNAs to knock down two core subunits, PSMA1 and PSMB1, in 2D10 cells and discovered that the KD potently reactivated latent HIV-1 and increased the viral *env* but not cellular GAPDH mRNA level (Fig 3A–3C).

Considering these results, we further tested whether inhibiting the proteasomal function with drugs could also reactivate latent HIV-1. We treated 2D10 and A2 cells with MG132, which is frequently used in research settings, three FDA approved proteasome inhibitors: Bortezomib (Millennium, Velcade, PS-341), Carfilzomib (ONYX, PR171), Ixazomib (Millennium, MLN2238), as well as three inhibitors in late stage clinical trials: Ixazomib citrate (MLN9708), Oprozomib (ONYX, ONX0912), Delanzomib (CEP18770). The results show that when used at nano- to submicro-molar concentrations, all the inhibitors were able to dose-dependently increase the HIV-1 LTR-driven transcription and reverse viral latency in up to ~80% of 2D10 and ~30% of A2 cells (Fig 3D–3I). Notably, these drug concentrations only mildly affected cell viability and no more than 50% cell death was observed even under the highest concentrations used (S3A–S3D Fig). Consistently, downregulating individual proteasome subunits by CRISPRi or shRNA for 3–5 days was in general fairly tolerated, although the loss of PSMD1 or PSMD3 produced a more prominent effect on cell viability (45% and 59% viable cells, respectively) compared to the loss of PSMD8, PSMA1 or PSMB1 (73%, 92% and 91%, respectively; S3E & S3F Fig). Together, our data indicate that targeting the proteasome by either gene silencing or drugs can effectively promote HIV-1 transcription and latency reversal.

### Proteasome inhibitors cooperate with existing LRAs to reactivate latent HIV-1 *ex vivo* without inducing T cell activation or proliferation

**Fig 4:**
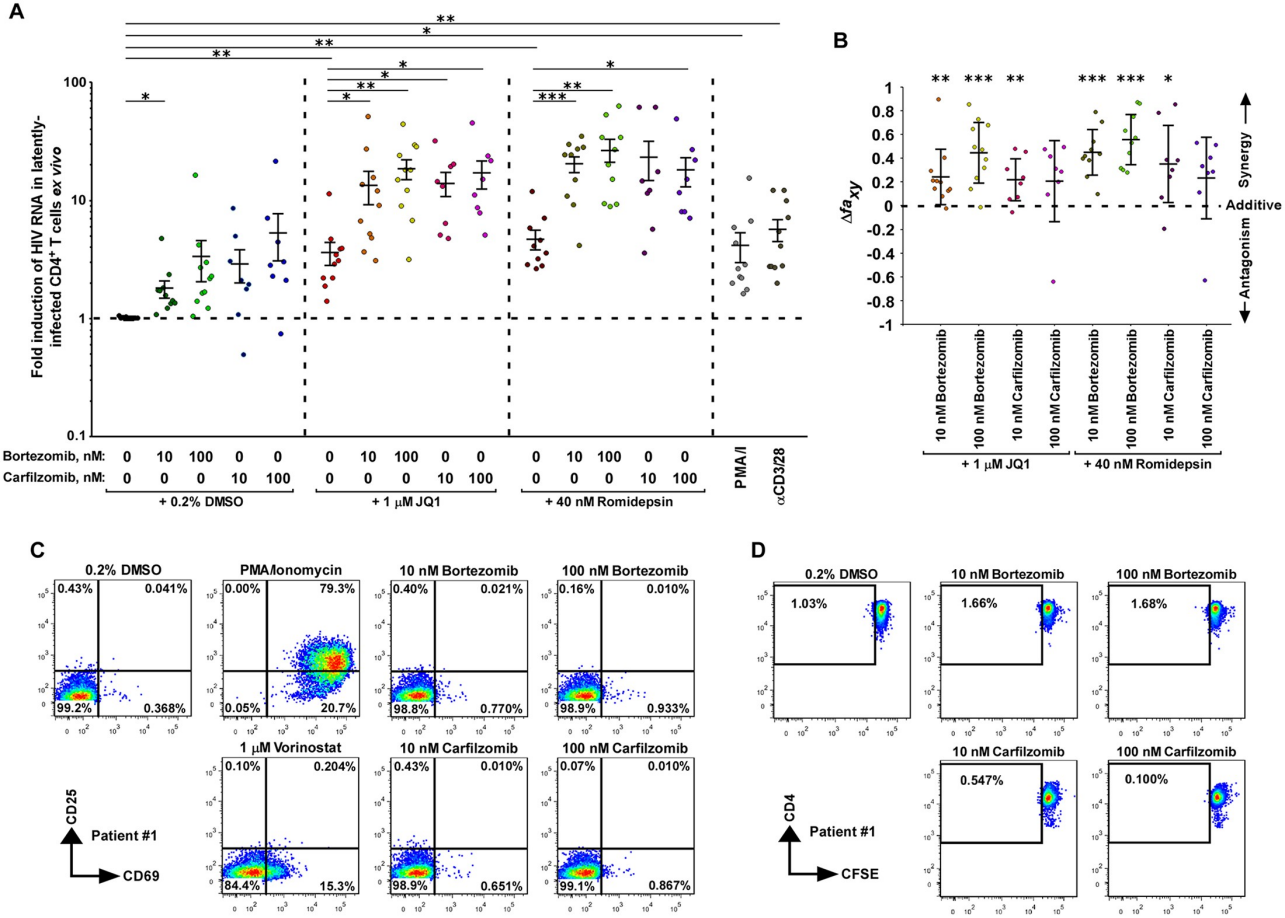
Proteasome inhibitors cooperate with existing LRAs to reactivate latent HIV-1 *ex vivo* without inducing T cell activation or proliferation. **A**. Freshly isolated CD4^+^ T cells from ART-suppressed HIV-1-infected individuals were treated with the indicated drug(s) for 24 hr. HIV-1 RNAs in the cells were quantified with RT-qPCR. The PCR signal from each drug combination was normalized to the DMSO group for each individual to calculate the fold induction displayed in the scatter plot. Mean ± standard error of the mean (SEM) is displayed, and the asterisks indicate the levels of statistical significance calculated by two-tailed unpaired t-tests (*: p<0.05, **: p<0.01, and ***: p<0.001). **B**. The Bliss independence model was used to assess drug synergism displayed by the indicated drug combinations. The mean ± standard deviation (SD) is shown for each combination. The asterisks indicate the levels of statistical significance calculated by two-tailed unpaired t-tests to compare between the predicted and observed drug combination effects (*: p<0.05, **: p<0.01, and ***: p<0.001). The dotted horizontal line denotes pure additive effect (Δ*fa*_*xy*_ = 0). Δ*fa*_*xy*_ > 0 indicates synergy, whereas Δ*fa*_*xy*_ < 0 indicates antagonism. **C**. Primary CD4^+^ T cells isolated from patient #1 were treated with the indicated drugs for 24 hr. The cell surface expression of the T cell activation markers CD69 and CD25 was examined by immunostaining and flow cytometry. **D**. Primary CD4^+^ T cells from patient #1 were stained with CellTrace CFSE, treated with the indicated drugs for 24 hr, cultured under drug-free conditions for 3 additional days, stained with the anti-CD4 fluorescent antibody, and then analyzed by flow cytometry.

To investigate the impact of inhibiting the proteasome in a more clinically relevant setting, we selected the two FDA-approved proteasome inhibitors, bortezomib and carfilzomib, and assessed their abilities to reactivate latent HIV-1 in CD4^+^ T cells isolated from 11 HIV-1-infected individuals on suppressive ART (S1 Table). While 10 nM bortezomib alone was able to reactivate HIV-1 by ~2-fold, the more significant finding is that both inhibitors potently enhanced the latency-reversing effects of existing LRAs at concentrations (10–100 nM; Fig 4A) that were effective in anti-cancer treatments [38,39].

For example, when used alone, 1 μM JQ1, a BET bromodomain inhibitor and known LRA [19,23], reactivated HIV-1 just 4-fold compared to the DMSO control. However, bortezomib and carfilzomib enhanced this effect up to 19-fold (Fig 4A). Furthermore, 40 nM of the HDAC inhibitor romidepsin [40,41] reactivated HIV-1 5-fold by itself, but produced up to 27-fold activation together with bortezomib or carfilzomib (Fig 4A). Finally, while 1 μM of the HDAC inhibitor vorinostat (aka SAHA; [42]) alone did not activate HIV-1 in a statistically significant manner, its combination with bortezomib or carfilzomib caused up to 11-fold activation (S4 Fig). Notably, the PKC agonist bryostatin [43] produced no obvious effect either alone or together with the two proteasome inhibitors (S4 Fig).

Using the Bliss Independence model for assessing drug synergism [21,24,44], we discovered that the co-administration of bortezomib (both 10 and 100 nM) or carfilzomib (10 nM) with JQ1 (1 μM) or romidepsin (40 nM) all exhibited robust synergistic activity (p<0.05) (Fig 4B). In addition, after 24 hours of treatment at 10 and 100 nM concentrations, which were the same conditions used in the latency-reversal assay, bortezomib and carfilzomib did not induce the surface expression of CD25 and only marginally induced CD69 in cells from 3 patients (Fig 4C & S5 Fig). This contrasts with the robust induction of the two activation markers by PMA plus ionomycin as well as the considerable CD69 induction by vorinostat (Fig 4C & S5 Fig).

Furthermore, staining with CellTrace CFSE detected no proliferation of live primary CD4^+^ T cells after the initial 24-hour exposure to the proteasome inhibitors and then additional 3 days of culture in the absence of the drugs (Fig 4D & S6 Fig). Finally, at least during the initial 24-hour treatment, the two inhibitors were well-tolerated by cells from all 3 patients, with the cells from patient #1 showing only a mild loss of viability in the presence of 10 nM carfilzomib for up to 4 days (S7 Fig). In summary, these data demonstrate that the proteasome inhibitors can synergize with existing LRAs to potently reactivate HIV-1 *ex vivo* without inducing activation or proliferation of the patient-derived primary CD4^+^ T cells.

### Inhibition or downregulation of proteasome increases Tat-transactivation by stabilizing ELL2 to form more ELL2-SECs

**Fig 5:**
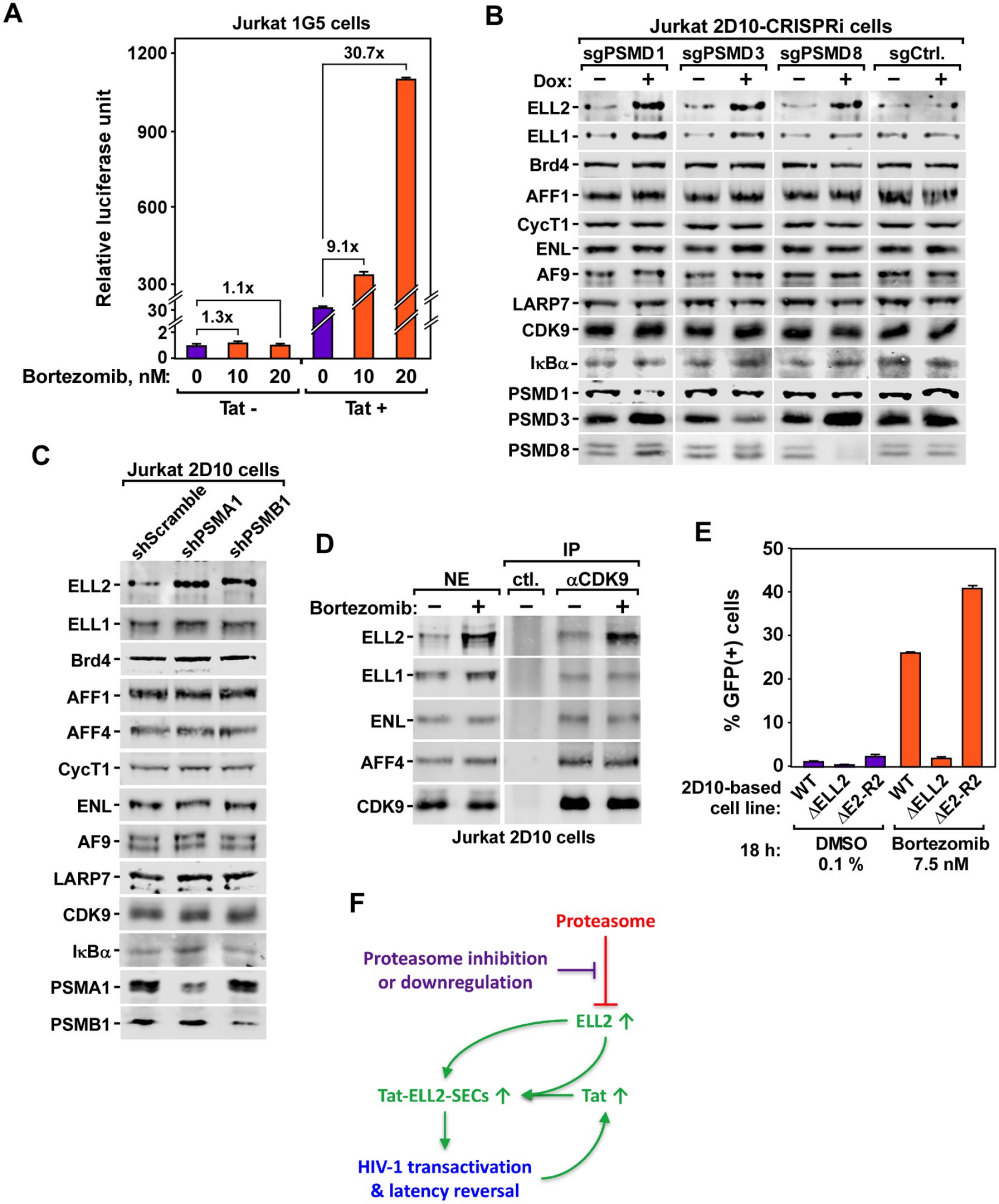
Inhibition or downregulation of proteasome increases Tat-transactivation by stabilizing ELL2 to form more ELL2-SECs. **A**. The Jurkat-based 1G5 and 1G5+Tat cells containing an integrated HIV-1 LTR-luciferase reporter construct were treated with the indicated concentrations of bortezomib or 0.1% DMSO for 20 hr. Luciferase activities in cell extracts were measured and shown. **B. & C**. Results of Western blot analyses of the effects of CRISPRi- or RNAi-induced downregulation of the indicated proteasome subunits in 2D10 cells on levels of the various proteins labeled on the left. **D**. Nuclear extracts (NE) were prepared from 2D10 cells treated with 10 nM bortezomib or 0.1% DMSO (-) for 18 hr and then subjected to immunoprecipitation (IP) with the anti-CDK9 antibody or rabbit total IgG as a control (ctl.). The NE and IP eluates were examined by Western blotting for the indicated proteins. **E**. Result of FACS analysis of the effects of treatment with 7.5 nM bortezomib or 0.1% DMSO for 18 hr on HIV-1 activation in three different 2D10-based cell lines: WT, ΔELL2 (ELL2-knockout) and ΔELL2-R2 (ΔELL2 cells containing an integrated vector expressing ELL2-Flag to about the endogenous level). **F**. A model showing the proposed mechanism by which the inhibition or downregulation of the proteasome stabilizes ELL2 to form more ELL2-SECs for HIV-1 Tat-transactivation and latency reversal. In A and E, error bars represent mean +/- SD from three experimental replicates.

Consistent with the CRISPRi result in Fig 2E, the bortezomib inhibition of the proteasome also enhanced the HIV-1 LTR-driven transcription in a Tat-dependent manner (Fig 5A). HIV-1 transcriptional elongation, especially the Tat-activated process, is exquisitely controlled by a network of P-TEFb complexes that include the 7SK snRNP, the SECs and the Brd4-P-TEFb complex [45]. In light of this revelation, we examined whether the levels of P-TEFb, its major known associated factors as well as the NFκB-inhibitor IκBα, which is believed to be regulated at the protein stability level [46,47], would change after the downregulation of the proteasome.

Examination of cell extracts by Western blotting demonstrates that among all the proteins analyzed, downregulating the proteasome in Jurkat cells by CRISPRi against PSMD1, PSMD3, and PSMD8 (Fig 5B), or RNAi against PSMA1 and PSMB1 (Fig 5C) consistently elevated the protein levels of only ELL2 and occasionally ELL1 (e.g. after CRISPRi against PSMD1 & PSMD3), which are two alternative subunits of the SECs [48]. Notably, the mRNA level of ELL2 was not elevated, but the ELL1 mRNA level was somewhat increased in this process (S8 Fig).

The elevated ELL2 protein level as a result of the proteasomal downregulation is consistent with the previous reports showing that ELL2 is tightly controlled by the E3 ubiquitin ligase Siah1-induced degradation by the proteasome [11,26,27]. Of note, inhibiting the proteasome by bortezomib also elevated the ELL2 protein level in Jurkat nuclear extract, which in turn resulted in the formation in the nuclei of more ELL2-containing SECs as revealed by anti-CDK9 immunoprecipitation followed by Western blotting (Fig 5D).

Since among all the related members of the family of SEC complexes, the ELL2-containg SECs play a predominant role in supporting Tat-transactivation and reversing viral latency [25], we compared the bortezomib-induced HIV-1 activation in three different 2D10-based cell lines: WT [29], ΔELL2 (ELL2-knockout) and ΔELL2-R2 (ΔELL2 cells containing an integrated vector expressing ELL2-Flag to approximately the endogenous level) [25]. The FACS analysis demonstrates that compared to WT 2D10 cells, the absence of ELL2 in ΔELL2 cells abolished the bortezomib-induced HIV-1 latency reversal, which was efficiently rescued by expressing ELL2-Flag in the ΔELL2-R2 cells (Fig 5E). Taken together, these results indicate the stabilization of ELL2 and elevated formation of the ELL2-SECs as a key mechanism for promoting HIV-1 Tat-transactivation and latency reversal in CD4^+^ T cells upon the inhibition/downregulation of the proteasome (Fig 5F).

## Discussion

In this study, we have developed a CRISPRi-based screen to reiteratively enrich loss-of-function genotypes that promote HIV-1 transcription in latently infected CD4^+^ T cells. The identified hits include the not-so-surprising factors that suppress the NF-κB pathway (NFKBIA, CYLD) or interact with the HDAC complex (GON4L), as well as three unexpected proteasomal subunits. Our subsequent experiments employing RNAi to target these three and also two other core subunits of the proteasome and testing various proteasome inhibitors in two different cell line-based latency models as well as primary CD4^+^ T cells from HIV-infected individuals on suppressive ART all support the notion that targeting the proteasome is an effective strategy to expose latent HIV-1.

Interestingly, a study published in 2004 has shown that the mRNA levels of multiple genes encoding the various proteasome subunits are upregulated in latently-infected cell lines and that treating these cells with a proteasome inhibitor CLBL stimulated lytic viral replication [49]. Based on these early revelations and our current study, which employs multiple proteasome inhibitors and extends the analysis to primary CD4^+^ T cells, we propose that the elevated proteasome level in HIV-infected cells is a key mechanism used to silence viral transcription and drive the virus into latency.

Consistent with a previous report showing that the proteasome inhibitors can enhance the P-TEFb-mediated HIV-1 transcriptional elongation [50], our current study pinpoints ELL2, which joins P-TEFb, AFF1 and ENL/AF9 to form the ELL2-SECs especially important for Tat-transactivation [11,25], as the target of the proteasome inhibitors. This insight as well as the observation that the Tat-dependent HIV-1 transcription is preferentially affected by targeting the proteasome (Figs 2E, 2F & 5A) allow us to propose a model in Fig 5F. According to this model, in latently infected cells, the elevated proteasome level keeps the ELL2 concentration low through polyubiquitination and proteasomal degradation [26]. This prevents the assembly of the ELL2-SECs and blocks HIV-1 transcription. Upon downregulating/inhibiting the proteasome, the blockage is removed to increase the cellular ELL2 level. This results in the formation of more ELL2-SECs to stimulate Tat-transactivation, which in turn generates more Tat to fuel a robust positive feedback loop for HIV to exit latency.

We have recently shown that the poly-ADP-ribosylation enzyme PARP1 upregulates ELL2 expression through inhibiting transcription as well as inducing degradation of Siah1 [27], the demonstrated E3 ubiquitin ligase for ELL2 [26]. During the control of HIV-1 transcription, the PARP1-Siah1 axis and the proteasome display strong similarities: Both preferentially affect the Tat-dependent transactivation process, and both accomplish this by controlling the cellular levels of ELL2 and ELL2-SECs. Because the PARP1-Siah1 axis works upstream of the proteasome-dependent regulation of ELL2 [27], it is tempting to speculate that by simultaneously augmenting PARP1 function and inhibiting the proteasome, it is possible to synergistically reactivate latent HIV-1, a hypothesis that is worth testing in future studies in primary CD4^+^ T cells.

The proteasome has been extensively characterized as a therapeutic target for treating both hematologic and solid tumors; and a number of inhibitors have been developed and approved for this purpose [51,52]. Our present study indicates that in addition to their anti-cancer effects, the two FDA-approved proteasome inhibitors, bortezomib and carfilzomib, can also synergize with existing LRAs such as JQ1 and romidepsin to reverse HIV latency in resting CD4^+^ T cells from ART-suppressed individuals without inducing T-cell activation or proliferation (Fig 5). Future studies will inform us whether this effect can also be detected in real clinical settings involving HIV patients. Moreover, the safety and efficacy of combining the proteasome inhibitors with other LRAs to expose the latent HIV-1 reservoirs for eradication also await further evaluation.

It is known that the proteasome regulates CD4^+^ T cell activation and proliferation through controlling cellular levels of various cyclins and cyclin-dependent kinase inhibitors, and that inhibiting proteasomal activity suppresses essential functions of activated CD4^+^ T cells [53,54]. In addition, the proteasome also modulates fate specification of CD8^+^ T cells during differentiation. Inhibiting the proteasome increased the number of effector CD8^+^ T cells and reduced the proportion of memory CD8^+^ T cells, and the inhibitor-treated CD8^+^ T cells exhibited increased killing of target cells in cytotoxicity assays [55,56]. Thus, proteasome inhibitors may suppress undesired CD4^+^ T-cell activation induced by other LRAs in HIV-infected individuals and promote killing of infected cells by CD8^+^ cells at the same time. Future studies will be needed to investigate the immunologic ramifications of proteasome inhibition in HIV-infected individuals.

Methodologically, the REACT protocol described here represents a significantly improved strategy to identify authentic genotypes that are hidden in a noisy background. Due to the stochastic nature of HIV-1 transactivation [57,58], the GFP-based HIV-1 latency models always display a small percentage of GFP-positive cells due to a low level of spontaneous viral activation [29,34]. This background noise could potentially mask and overwhelm the real signals in any genome-wide screens that must start with a pooled library. The complexity of such libraries causes each genotype to have an extremely low representation in the whole population. Therefore, the phenotypic change induced by a to-be-identified genotype in only a few cells, even though genuine and significant, could easily be lost in a noisy background as exemplified by the first two rounds of REACT in our study. Only through repeated cycles of enrichment, the desired genotypes can be progressively enriched and become prominent in the population as demonstrated by high throughput sequencing of the sgRNA libraries enriched from round 1 to 4 of REACT (Fig 1B and S1 Fig). Thus, although REACT may under-sample genotypes that inhibit cell growth, it can still be very useful for identifying the genetic basis of other noisy phenotypes that are not amenable to the single-round genome-wide screens.

## Materials and methods

### Ethics statement

The part of this study utilizing specimens from HIV-infected individuals was approved by the UCSF Committee on Human Research. All research participants were recruited from the UCSF SCOPE cohort after obtaining written informed consent, and all subject data and specimens were coded to protect confidentiality. All participants were adults and met strict selection criteria and had well-documented persistent viral suppression for over 7 years (S1 Table).

### Reiterative Enrichment and Authentication of CRISPRi Targets (REACT)

All the Jurkat-based cells were maintained in RPMI 1640 medium with L-glutamine, 10% fetal bovine serum (FBS), 100 IU/ml penicillin, and 100 μg/ml streptomycin in 5% CO_2_ at 37°C. To prepare the CRISPR interference (CRISPRi) platform, Jurkat 2D10 cells (previously generated by Karn lab based on human CD4^+^ T cells Jurkat line [29]) were transduced by pHR-TRE3G-Krab-dCas9-P2A-mCherry [30] and pLVX-advanced-TetOn (from IGI, UC Berkeley). A clone (named “2D10-CRISPRi”) expressing KRAB-dCas9-HA-P2A-mCherry in response to doxycycline (Dox) was first selected by fluorescence-activated cell sorting (FACS) and then verified by Western blotting [59].

To start REACT, a genome-wide CRISPRi sgRNA library in pSico-based vector with BFP marker and puromycin-resistance [30] were packaged using a 3^rd^ generation lentiviral packaging system and transduced into 2×10^8^ 2D10-CRISPRi cells at an efficiency of ~40%. Two days after transduction, the non-transduced cells were killed by adding puromycin into the medium to a final concentration of 1 μg/ml for 3 days, at which time more than 95% of the surviving cells were BFP-positive as confirmed by FACS. About 5×10^7^ of the cells were pooled and treated with 1 μg/ml Dox for 3 days in 400 ml medium. The Dox-treated cells were then selected by FACS for the GFP/mCherry/BFP triple-positive phenotype.

Then sgRNA cassettes were PCR-amplified from the genomes of the selected cells using the primer pair: REACT-5F (5’-GCACAAAAGGAAACTCACCCT-3’)/ REACT-3R (5’-CGACTCGGTGCCACTTTTTC-3’). After digestion with BstXI and BlpI, the cassettes were cloned into the empty library vector pSico-BFP-puro [30] and then amplified in *E*. *coli* and extracted as an enriched library, which was then transduced into the original 2D10-CRISPRi cells for the next round of REACT. Upon repeating the procedure 4 times, the sgRNA sequences from the enriched libraries and original library were amplified by using the index primer pairs CRISPRi_TSS_12_P5/CRISPRi_TSS_12_P7 or CRISPRi_TSS_6_P5/CRISPRi TSS_6_P7, and deep-sequenced by using the primer CRISPRi TSS_seq V2. The sequences of the primers were listed in S2 Table. The deep-sequencing results were then converted into sgRNA counts by using the ScreenProcessing tool [60]. The fold of enrichment for each sgRNA sequence was calculated based on its reads per million in the round 4-enriched library divided by those in the original library and presented on a scatter plot.

### Cell line-based latency reversal assay

The Jurkat-based HIV-1 latency models 2D10 (previously generated by Karn lab based on human CD4^+^ T cells Jurkat line [29]) and J-Lat A2 (previously generated by Verdin lab based on human CD4^+^ T cells Jurkat line [34]) were first treated in triplicates with 0.1% DMSO, 1 μg/ml Dox or the various concentrations of proteasome inhibitors, and then re-suspended in cold phosphate-buffered saline (PBS). Quantification of the GFP+ cells was conducted on a BD Bioscience LSR Fortessa X20 cytometer. The data were analyzed with the Flowjo software and plotted as bar graphs.

### CRISPRi in 1G5-/+Tat cells and luciferase reporter assay

The preparations of the CRISPRi platform in Jurkat 1G5 [35] and 1G5+Tat cells [36] (both kind gifts from Dr. Melanie Ott in the Gladstone Institutes, San Francisco) were the same as in 2D10 cells described above. DNA oligos containing the sgRNA sequences identified by REACT and a negative control (5’-GCAGCATGCTCGCCTGGCTGC-3’) were synthesized and cloned into the pSico-BFP-puro vector and stably transduced into the 1G5-/+Tat-CRISPRi cells. For the luciferase assay, 1x10^6^ of the cells were treated with 0.1% DMSO or 1 μg/ml Dox in triplicates for three days, and then lysed in 200 μl 1 × Reporter Lysis Buffer (Promega), frozen-thawed once, and centrifuged at 20,800 × g for 1 min at 4 °C. Luciferase activities in the supernatants were measured by using the Luciferase Assay System (Promega) on a Lumat LB 9501 luminometer. The relative luciferase units in the Dox-treated cells were normalized to those of the DMSO-treated cells and plotted as bar graphs.

### Reverse transcription and real-time quantitative PCR (RT-qPCR) assay

Total cellular RNAs were extracted by using the RNeasy kit (Qiagen) and reverse transcribed by M-MLV Reverse Transcriptase (VWR, M1701) with random hexamers (Invitrogen, 48190–011). The cDNAs were subjected to qPCR using DyNAmo HS SYBR Green qPCR kit (Fisher, F-410L) on a CFX96 system (Bio-Rad) with the primer pairs listed in S2 Table. All reactions were carried out in triplicates. The PCR signals were normalized to those of ActB and displayed as bar graphs.

### Stable shRNA knockdown (KD) in Jurkat 2D10 cells

Jurkat 2D10 cells were transduced with the pLKO.1-puro lentiviral vectors containing shScramble (5’-CCTAAGGTTAAGTCGCCCTCG-3’), shPSMA1 (5'-AATGATTATACAGACCCTTTC-3'), or shPSMB1 (5’-AAGACATCTTTCACCAGCCGC-3’) sequences. Two days after transduction, puromycin was added to the medium to a final concentration of 1 μg/ml to obtain and maintain the stable KD pools. The KD efficiencies of the pools were examined by RT-qPCR and Western blot.

### Proteasome inhibitor treatment *in vitro*

Jurkat 2D10 or J-Lat A2 cells were aliquoted into 24-well plates in 1 ml medium at the density of 5×10^5^ cells/ml. Each of the following drugs was added to the cells in triplicate at the indicated concentrations: MG132 (Sigma, M7449), Bortezomib (Millennium, Velcade, PS-341, from Selleckchem, S1013), Carfilzomib (ONYX, PR171, from Selleckchem, S2853), Ixazomib (Millennium, MLN2238, from Selleckchem, S2180), Ixazomib citrate (MLN9708, from Selleckchem, S2181), Oprozomib (ONYX, ONX0912, from Selleckchem, S7049), and Delanzomib (CEP18770, from Selleckchem, S1157). For control groups, 0.1% DMSO was used. After 16~20 hours treatment, the cells were subjected to FACS to measure the percentages of GFP+ cells as described above. Cell viabilities were determined by Forward Scatter vs. Side Scatter gating using untreated cells as the control.

### Co-immunoprecipitation followed by Western blotting

Approximately 2 × 10^8^ 2D10 cells maintained in 400 ml RPMI medium were treated with 10 nM bortezomib or 0.1% DMSO for 18 hours. The following operations were all carried out at 4 °C or on ice unless stated otherwise. The cells were harvested and swollen in 4 ml hypotonic buffer A [10 mM HEPES-KOH (pH 7.9), 1.5 mM MgCl_2_ and 10 mM KCl] for 5 min and then centrifuged at 362 g for 5 min. The cells were then disrupted by grinding 20 times with a Dounce tissue homogenizer (Fisher K8853000007) in 4 ml buffer A, followed by centrifugation at 3,220 g for 10 min to collect the nuclei. The nuclei were then extracted in 450 μl buffer C [20 mM HEPES-KOH (pH 7.9), 0.42 M NaCl, 25% glycerol, 0.2 mM EDTA, 1.5 mM MgCl_2_, 0.4% NP-40, 1 mM dithiothreitol and 1 × protease inhibitor cocktail] for 30 min and then subjected to centrifugation at 20,800g for 10 min. The supernatant (nuclear extracts or NE) was mixed with 4 μg anti-CDK9 antibody [11] for 45 min, and then with 15 μl Protein A agarose (Invitrogen 15918–014) for 1 hr with rotation. After being washed three times with 1 ml buffer D [20 mM HEPES-KOH (pH 7.9), 0.3 M KCl, 15% glycerol, 0.2 mM EDTA and 0.4% NP-40], the beads were eluted with 30 μl 0.1 M glycine-HCl (pH 2.0) at room temperature for 15 min. For western blot, 3% of the NE input and 50% of the immunoprecipitation eluate from each treatment condition were analyzed.

The primary antibodies used for Western blots are listed in S3 Table. The primary antibodies were diluted to 1 μg/ml and the secondary antibodies were diluted 10,000-fold.

### Isolation of resting CD4^+^ T cells from ART-suppressed individuals and measurement of intracellular HIV-1 mRNA after drug treatment

Fresh blood (100 ml) was collected and peripheral blood mononuclear cells (PBMCs) were isolated from whole blood using Lymphocyte Separation Medium (Corning 25-072-CI). CD4^+^ T cells were isolated from PBMCs using negative selection by EasySep kit (STEMCELL 19052) according to the manufacturer’s instructions.

Isolated CD4^+^ T cells were aliquoted at a density of 1×10^6^ cells per well in 1 mL RPMI medium plus 10% FBS in a 48-well flat-bottom plate. The cells were treated with 0.2% DMSO, 25 μl (1:1 bead:cell ratio) αCD3 + αCD28-conjugated beads (Dynal 11131D), 50 ng/ml (81 nM) PMA + 1 μM Ionomycin, 1 μM vorinostat, 1 μM JQ1, 40 nM romidepsin, or 10 nM bryostatin alone or in combination with 10 nM or 100 nM bortezomib or carfilzomib for 24 hr. All drugs were prepared in the culture medium from stock solutions dissolved in DMSO. After the treatment, total RNAs from the cells were extracted by 1 ml TRIzol Reagent (Invitrogen 15596026). The RNAs were reverse-transcribed using M-MLV Reverse Transcriptase (VWR, M1701) with random hexamers (Invitrogen, 48190–011). The HIV-1 RNA was quantified by qPCR using DyNAmo HS SYBR Green qPCR kit (Fisher, F-410L) with the HIV-1-specific primers F522-43 (5’-GCCTCAATAAAGCTTGCCTTGA-3’) and R626-43 (5’-GGGCGCCACTGCTAGAGA-3’) [24] on a CFX96 system (Bio-Rad). All reactions were carried out in triplicates. The PCR signal from each drug combination was normalized to the DMSO group for each individual to calculate the fold induction and displayed in scatter plots.

### Quantitative analysis of synergy of latency reversing agent combinations

We adapted the Bliss independence model [44] as implemented by previous studies [21,22,24] to test for synergy when different concentrations of bortezomib and carfilzomib were combined with JQ1 or romidepsin *ex vivo*. For drugs x and y, we used the equations *fa*_*xyP*_ = *fa*_*x*_ *+ fa*_*y*_*—(fa*_*x*_*)(fa*_*y*_*)*, and Δ*fa*_*xy*_ = *fa*_*xyO*_*—fa*_*xyP*_. Here, *fa*_*x*_ and *fa*_*y*_ represent the observed effects of drug x and drug y respectively, *fa*_*xyP*_ represents the predicted fraction affected by the combination of drug x and drug y if there is no synergy or antagonism between drug x and drug y; *fa*_*xyO*_ represents the observed effect when x and y were tested together. Calculation of *fa*_*x*_ utilized the following approach adapted from the above cited publications: *fa*_*x*_ = (HIV RNA copies with drug x—background copies with DMSO) / (HIV RNA copies with αCD3-αCD28 stimulation—background copies with DMSO). The copy number of HIV RNA was determined by extrapolation against a 7-point standard curve (1–1,000,000 copies) prepared from a synthetic HIV cDNA fragment. In cases where one or more experimental drug conditions resulted in RNA expression exceeding the αCD3-αCD28 stimulation, we imputed the highest HIV RNA value in that experiment +1 to represent the denominator for calculation of *fa*_*x*_. Statistical significance was calculated by two-tailed Student’s t-test comparing *fa*_*xyO*_ and *fa*_*xyP*_ (*: p < 0.05, **: p < 0.01, and ***: p < 0.001). With this model, Δ*fa*_*xy*_ > 0 with statistical significance (p < 0.05) indicates synergy, Δ*fa*_*xy*_ = 0 indicates additive effect (Bliss independence), Δ*fa*_*xy*_ < 0 with statistical significance indicates antagonism.

### CD4^+^ T cell activation assay

CD4^+^ T cells isolated from HIV-infected ART-suppressed individuals were treated with 0.2% DMSO, 50 ng/ml (81 nM) PMA and 1 μM Ionomycin, 1 μM vorinostat, 10 nM bortezomib, 100 nM bortezomib, 10 nM carfilzomib, or 100 nM carfilzomib for 24 hours. The cells were stained with LIVE/DEAD Cell Stain Kit (Invitrogen, L34955), and then stained with PE-conjugated mouse anti-Human CD69 antibody (BD Biosciences, 555531), and FITC-conjugated mouse anti-Human CD25 antibody (BD Biosciences, 555431). Flow cytometry was conducted on a BD Bioscience LSR Fortessa X20 cytometer, and data were analyzed using the Flowjo software.

### CD4^+^ T cell proliferation assay

CD4^+^ T cells isolated from HIV-infected ART-suppressed individuals were stained with 10 μM 5(6)-carboxyfluorescein N-hydroxysuccinimidyl ester (CFSE, Abcam ab113853) for 15 min. The cells were treated subsequently with 0.2% DMSO, 10 nM bortezomib, 100 nM bortezomib, 10 nM carfilzomib, or 100 nM carfilzomib for 24 hr, and then washed and cultured for another 3 days in fresh medium. The cells were stained with LIVE/DEAD Cell Stain Kit (Invitrogen, L34955) and PE-conjugated anti-human CD4 Antibody (BioLegend, 317410). Flow cytometry and data analysis were conducted as described above.

### CD4^+^ T cell viability assay

CD4^+^ T cells isolated from HIV-infected ART-suppressed individuals were treated with the various drugs for 4 days as described above. On day 1, 2, 3, and 4, an aliquot of cells from each treatment was stained with LIVE/DEAD Cell Stain Kit (Invitrogen, L34955). Untreated cells were used for day 0. Flow cytometry and data analysis were conducted as described above.

## Supporting information

S1 Fig A progressive enrichment of the main hits from background noise during 4 rounds of REACT. **A., B., C., & D**. The sgRNA libraries enriched from round 1 to round 4 of REACT were subjected to high throughput sequencing and the fold of enrichment for each sgRNA was calculated based on its reads per million divided by those in the original library and presented on scatter plots. The genes targeted by the most significantly enriched sgRNAs in each library were labelled.
(PDF)

S2 Fig Verification of target specificity and HIV-1 activation potential of 3 selected REACT-identified genes by RNAi in Jurkat 2D10 and J-Lat cells. **A., B., C., D., E., & F**. Jurkat 2D10 (A, B, & C) or J-Lat A2 (D, E, & F) cells were nucleofected with siRNAs targeting the indicated genes or nothing (NT). Shown were results of FACS analyses of the GFP-expressing 2D10 (A) or J-Lat A2 (D) cells containing activated HIV-1. Results of RT-qPCR analyses of expression levels of the genes indicated by their corresponding qPCR primers in aliquots of 2D10 (B & C) or J-Lat A2 (E & F) cells were also shown. Error bars in all panels represent mean +/- SD from three experimental replicates. Asterisks denote levels of statistical significance calculated by two-tailed Student’s t-test (*: p<0.05, **: p<0.01, and ***: p<0.001).
(PDF)

S3 Fig Effects of inhibiting the proteasome or silencing the expression of its individual subunits on viability of Jurkat 2D10 and J-Lat cells. **A., B., C., & D**. Jurkat 2D10 (A & C) or J-Lat A2 (B & D) cells were treated with the indicated proteasome inhibitors at the described concentrations. **E. & F**. Indicated proteasome subunits were downregulated in Jurkat 2D10 cells by either CRISPRi or RNAi for 3 and 5 days respectively. Cell viabilities were determined by Forward Scatter vs. Side Scatter gating using untreated cells as the control. Error bars represent mean +/- SD from three experimental replicates. The data analyzed in this figure were from the same experiments in Figs 3D, 3F, 3H, 3I, 2B, and Fig 3A.
(PDF)

S4 Fig Effect of combining bortezomib or carfilzomib with vorinostat and bryostatin on HIV-1 transcriptional activation in latently infected CD4^+^ T cells from ART-suppressed individuals. Freshly isolated CD4^+^ T cells (same as in Fig 4) from ART-suppressed HIV-1-infected individuals were treated with the indicated drug(s) for 24 hr. HIV-1 RNAs in the cells were quantified with RT-qPCR. The PCR signal from each drug combination was normalized to that of the DMSO group (not shown here but same as in Fig 4) for each individual to calculate the fold induction displayed in the scatter plot. Mean ± SEM is displayed, with the asterisks indicating the levels of statistical significance compared with the DMSO group calculated by two-tailed unpaired t-tests (*: p<0.05, **: p<0.01, and ***: p<0.001).
(PDF)

S5 Fig Effects of proteasome inhibitors on T cell activation. **A. & B**. Primary CD4^+^ T cells isolated from ART-suppressed HIV-1-infected patient #2 (A) and #3 (B) were treated with the indicated drugs for 24 hr. The cell surface expression of CD69 and CD25 was examined by immunostaining and flow cytometry.
(PDF)

S6 Fig Effects of proteasome inhibitors on proliferation of primary CD4^+^ T cells. **A. & B**. Primary CD4^+^ T cells from ART-suppressed HIV-1-infected patient #2 (A) and #3 (B) were stained with CellTrace CFSE, treated with the indicated drugs for 24 hr, cultured under drug-free conditions for 3 additional days, stained with the anti-CD4 fluorescent antibody, and then analyzed by flow cytometry.
(PDF)

S7 Fig Effects of proteasome inhibitors on CD4^+^ T cell viability. **A., B., & C**. Primary CD4^+^ T cells isolated from ART-suppressed HIV-1-infected patient #1 (A), #2 (B) and #3 (C) were treated with the indicated drugs for 4 days. An aliquot of cells from each treatment was collected on the indicated days, stained with LIVE/DEAD Cell Stain Kit (Invitrogen, L34955), and subjected to flow cytometry to quantify the percentages of live cells.
(PDF)

S8 Fig Effect of downregulation of proteasome subunits on mRNA levels of ELL1 and ELL2. **A. & B**. Results of RT-qPCR analyses of the mRNA levels of ELL1 and ELL2 in aliquots of the cells treated and examined in Fig 5B & 5C. For each group, the mRNA level in the DMSO-treated cells was set to 1. Error bars represent mean +/- SD from three experimental replicates. Asterisks denote levels of statistical significance calculated by two-tailed Student’s t-test (*: p<0.05, **: p<0.01, and ***: p<0.001).
(PDF)

S1 Table Characteristics of HIV-1–infected study participants. (PDF)

S2 Table List of DNA oligonucleotide primers used in this study. (PDF)

S3 Table List of antibodies used in this study. (PDF)
