# Supplementary figures and images for: Reiterative Enrichment and Authentication of CRISPRi Targets (REACT) identifies the proteasome as a key contributor to HIV-1 latency
Source: PLoS Pathog. 2019 Jan 15;15(1):e1007498. doi: 10.1371/journal.ppat.1007498 (PMC6333332; doi:10.1371/journal.ppat.1007498)

**A**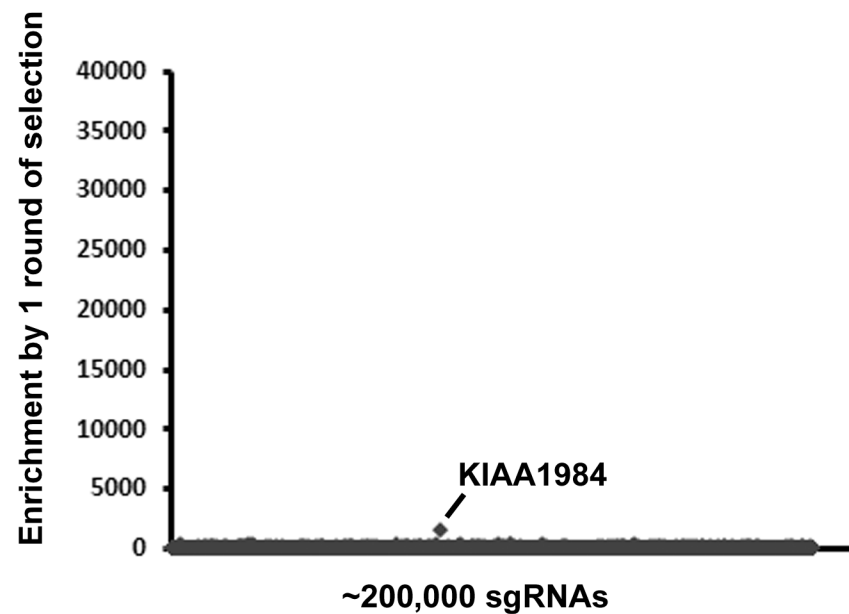**B**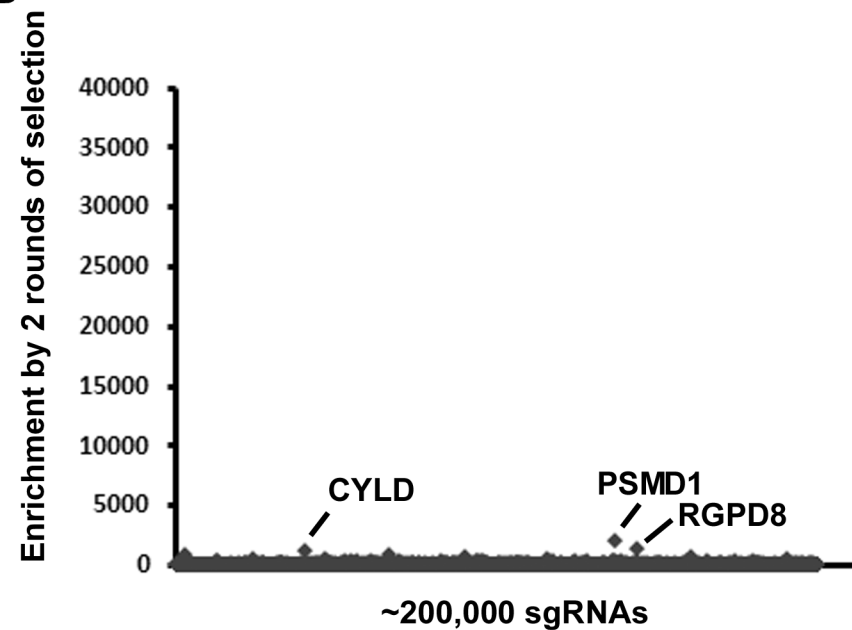**C**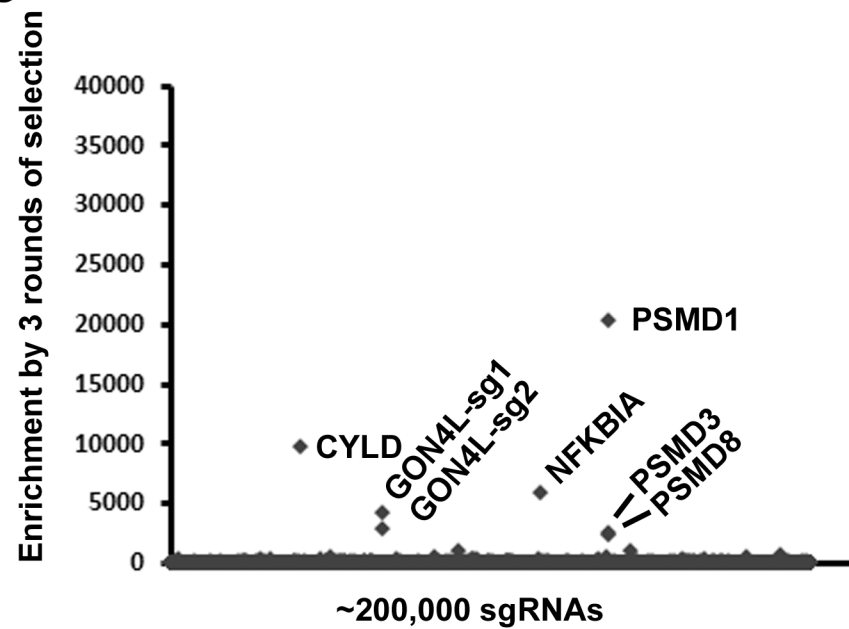**D**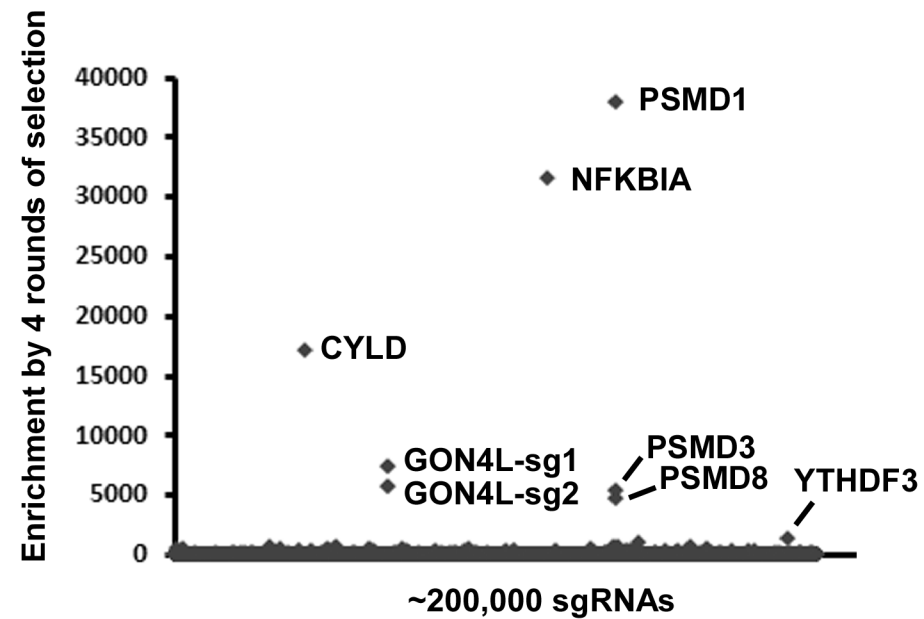

Supplement: S1 Fig — A., B., C., & D. The sgRNA libraries enriched from round 1 to round 4 of REACT were subjected to high throughput sequencing and the fold of enrichment for each sgRNA was calculated based on its reads per million divided by those in the original library and presented on scatter plots. The genes targeted by the most significantly enriched sgRNAs in each library were labelled. (PDF) [file ppat.1007498.s001.pdf]

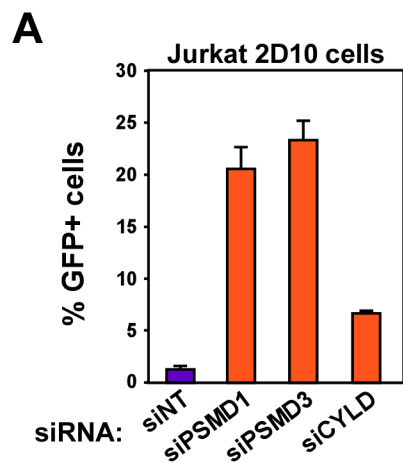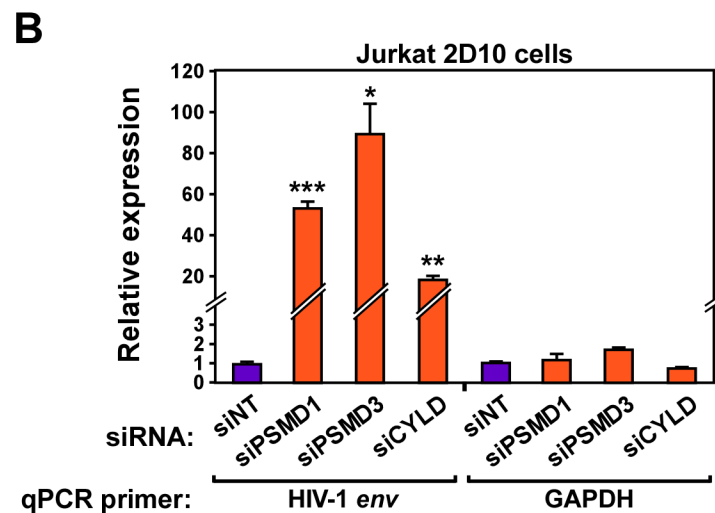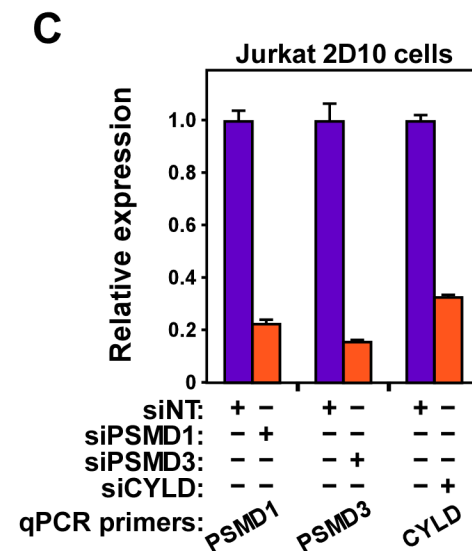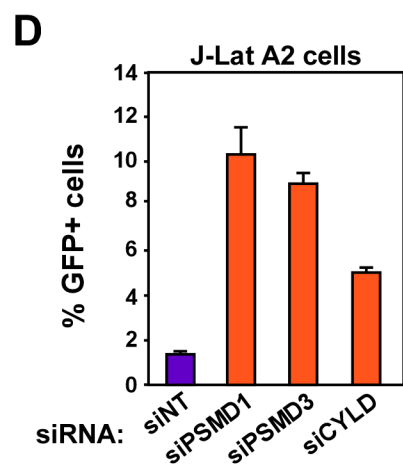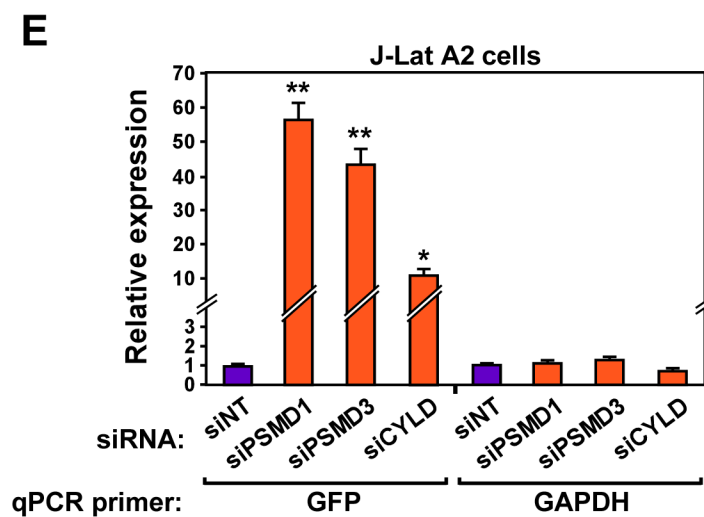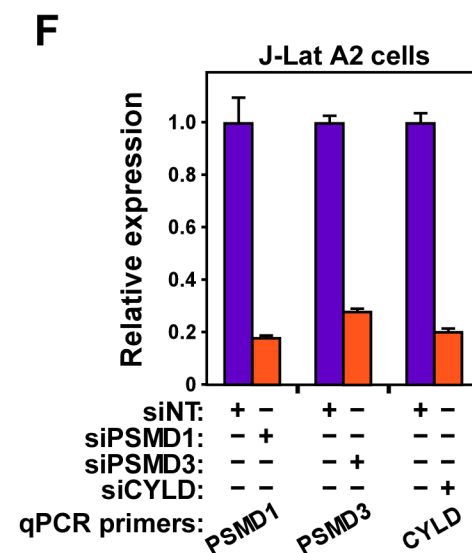

Supplement: S2 Fig — A., B., C., D., E., & F. Jurkat 2D10 (A, B, & C) or J-Lat A2 (D, E, & F) cells were nucleofected with siRNAs targeting the indicated genes or nothing (NT). Shown were results of FACS analyses of the GFP-expressing 2D10 (A) or J-Lat A2 (D) cells containing activated HIV-1. Results of RT-qPCR analyses of expression levels of the genes indicated by their corresponding qPCR primers in aliquots of 2D10 (B & C) or J-Lat A2 (E & F) cells were also shown. Error bars in all panels represent mean +/- SD from three experimental replicates. Asterisks denote levels of statistical significance calculated by two-tailed Student’s t-test (*: p<0.05, **: p<0.01, and ***: p<0.001). (PDF) [file ppat.1007498.s002.pdf]

**A**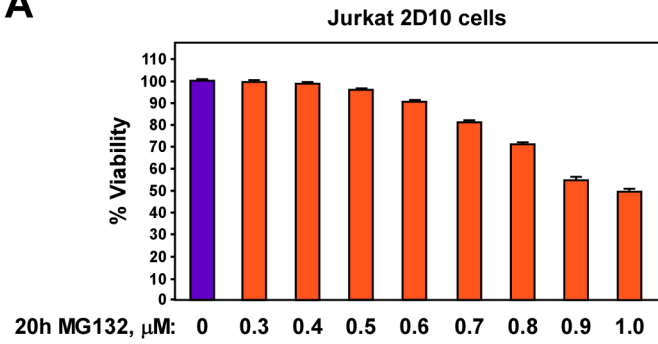**B**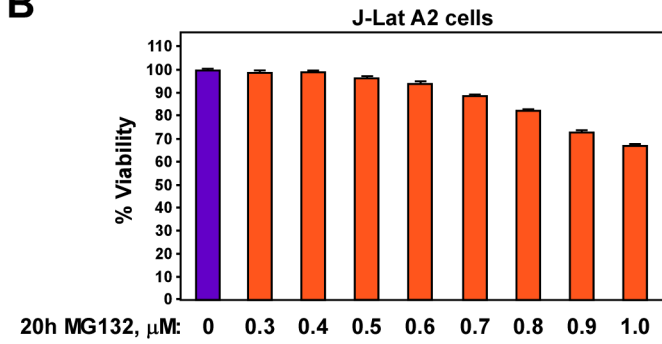**C**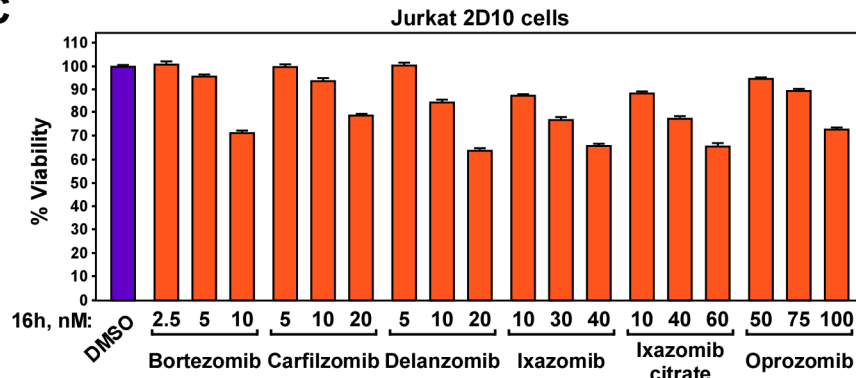**D**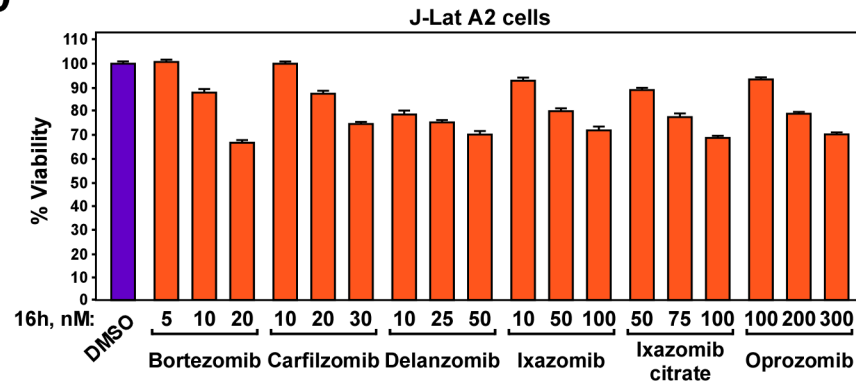**E**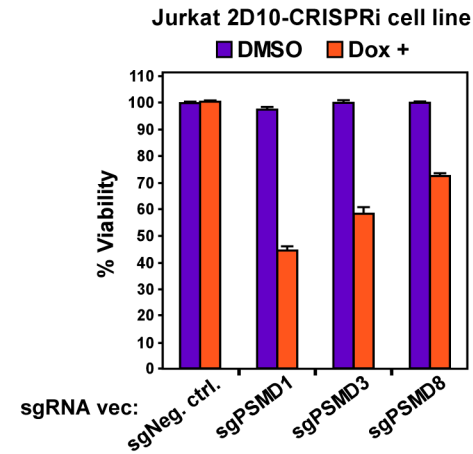**F**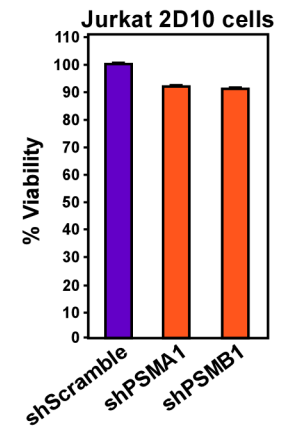

Supplement: S3 Fig — A., B., C., & D. Jurkat 2D10 (A & C) or J-Lat A2 (B & D) cells were treated with the indicated proteasome inhibitors at the described concentrations. E. & F. Indicated proteasome subunits were downregulated in Jurkat 2D10 cells by either CRISPRi or RNAi for 3 and 5 days respectively. Cell viabilities were determined by Forward Scatter vs. Side Scatter gating using untreated cells as the control. Error bars represent mean +/- SD from three experimental replicates. The data analyzed in this figure were from the same experiments in Figs 3D, 3F, 3H, 3I, 2B, and Fig 3A. (PDF) [file ppat.1007498.s003.pdf]

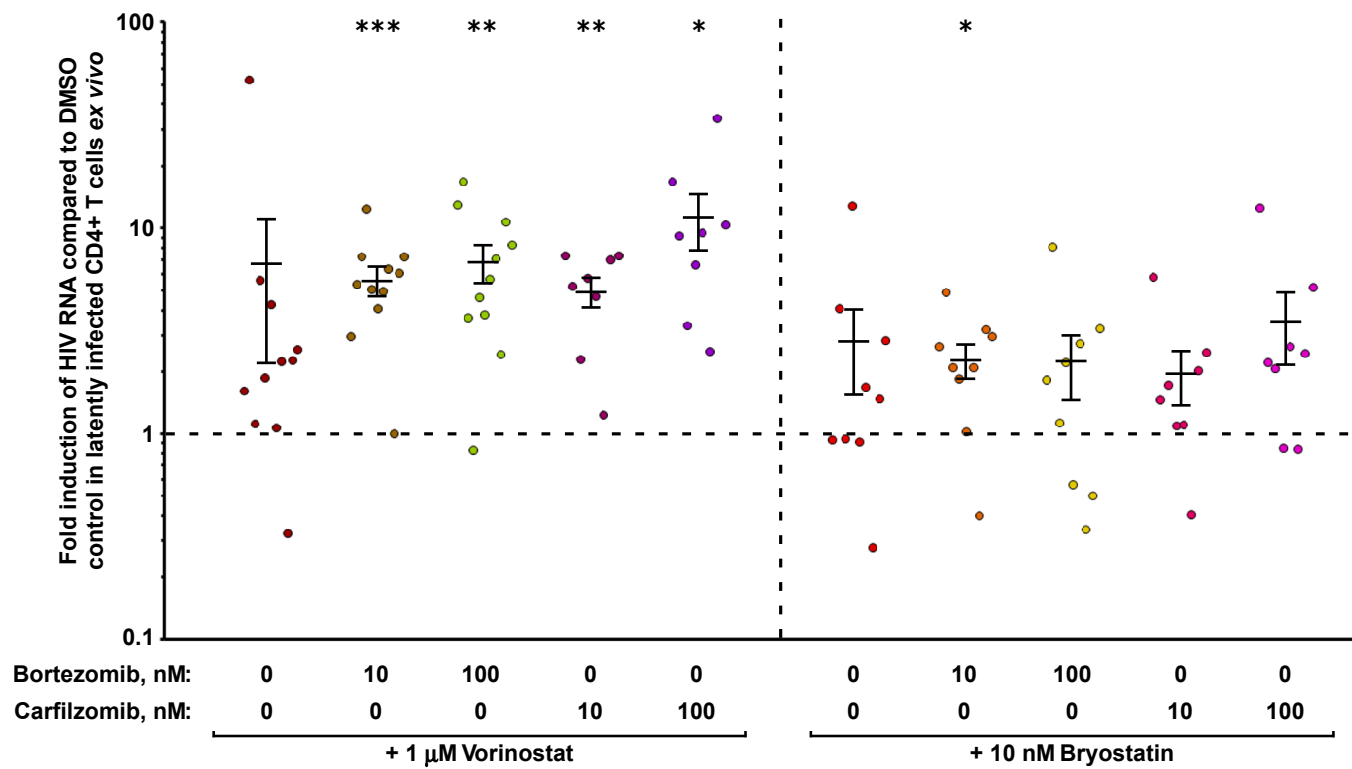

Supplement: S4 Fig — Freshly isolated CD4+ T cells (same as in Fig 4) from ART-suppressed HIV-1-infected individuals were treated with the indicated drug(s) for 24 hr. HIV-1 RNAs in the cells were quantified with RT-qPCR. The PCR signal from each drug combination was normalized to that of the DMSO group (not shown here but same as in Fig 4) for each individual to calculate the fold induction displayed in the scatter plot. Mean ± SEM is displayed, with the asterisks indicating the levels of statistical significance compared with the DMSO group calculated by two-tailed unpaired t-tests (*: p<0.05, **: p<0.01, and ***: p<0.001). (PDF) [file ppat.1007498.s004.pdf]

**A**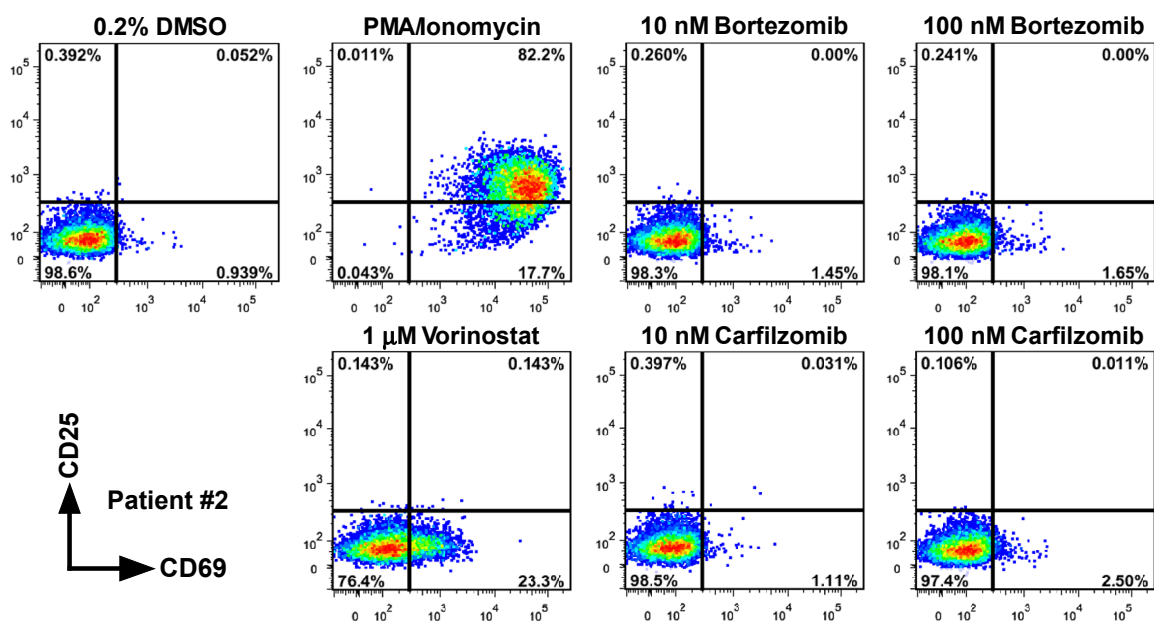**B**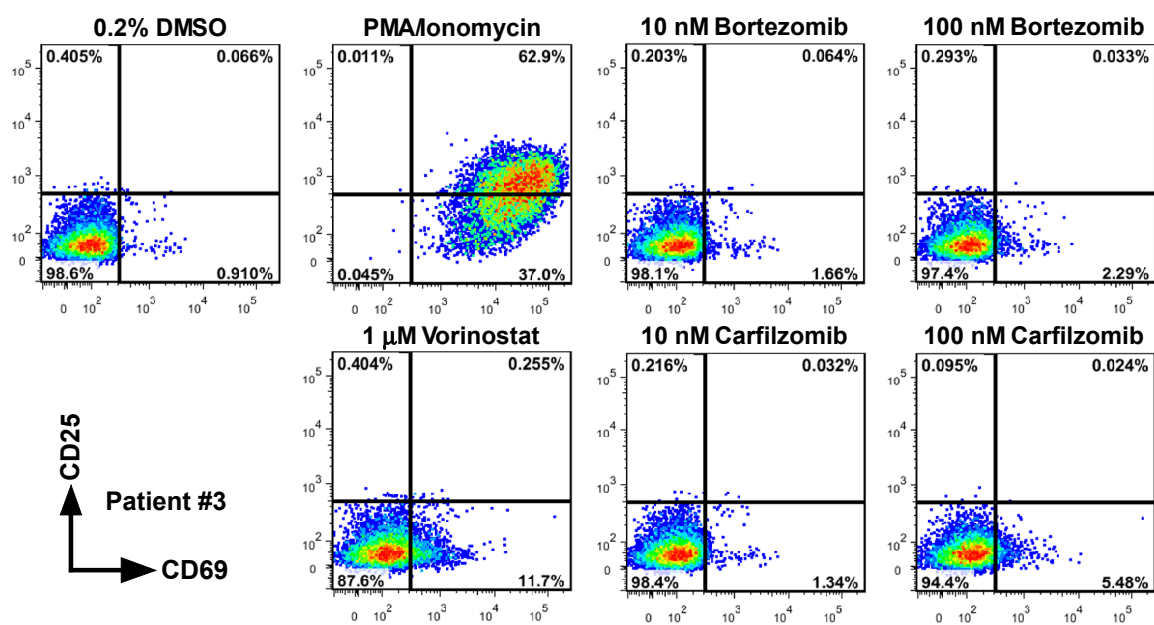

Supplement: S5 Fig — A. & B. Primary CD4+ T cells isolated from ART-suppressed HIV-1-infected patient #2 (A) and #3 (B) were treated with the indicated drugs for 24 hr. The cell surface expression of CD69 and CD25 was examined by immunostaining and flow cytometry. (PDF) [file ppat.1007498.s005.pdf]

**A**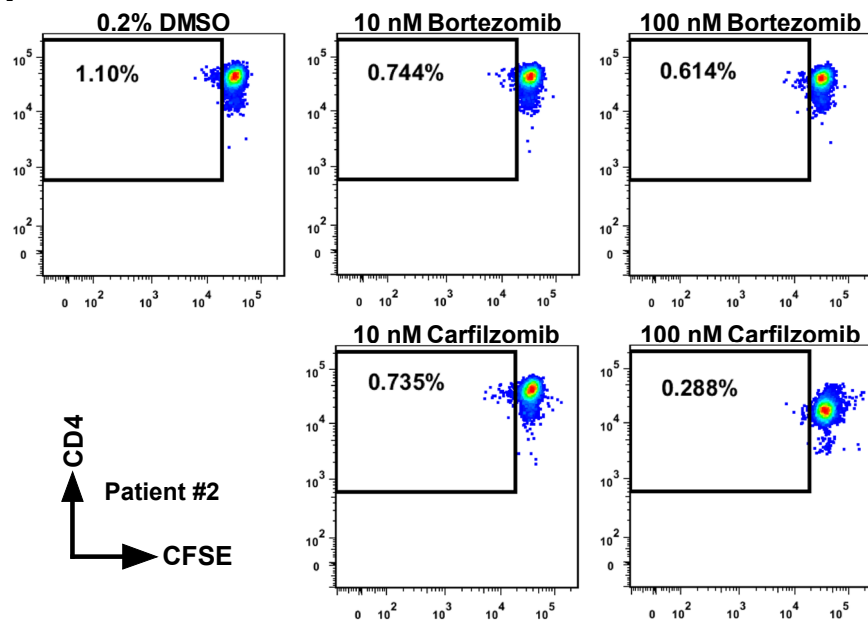**B**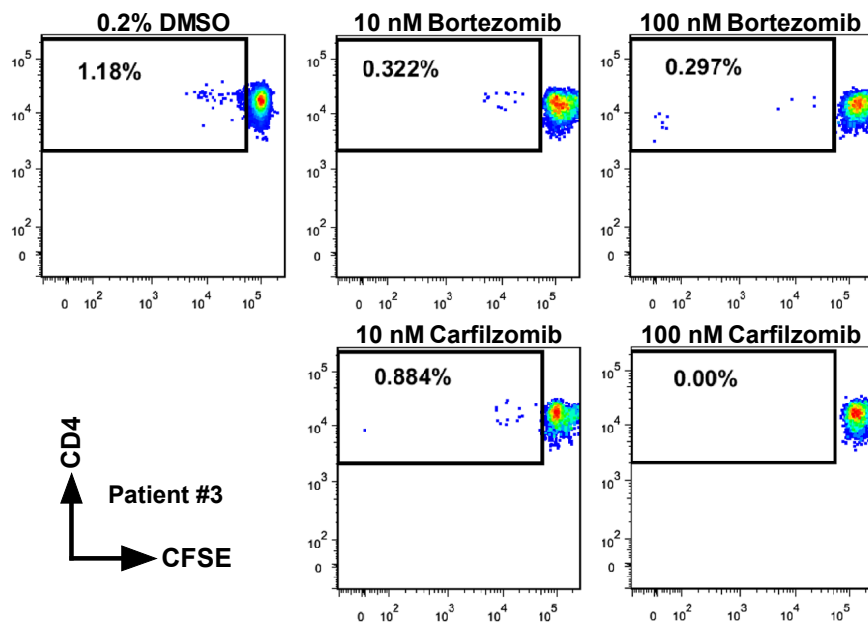

Supplement: S6 Fig — A. & B. Primary CD4+ T cells from ART-suppressed HIV-1-infected patient #2 (A) and #3 (B) were stained with CellTrace CFSE, treated with the indicated drugs for 24 hr, cultured under drug-free conditions for 3 additional days, stained with the anti-CD4 fluorescent antibody, and then analyzed by flow cytometry. (PDF) [file ppat.1007498.s006.pdf]

**A**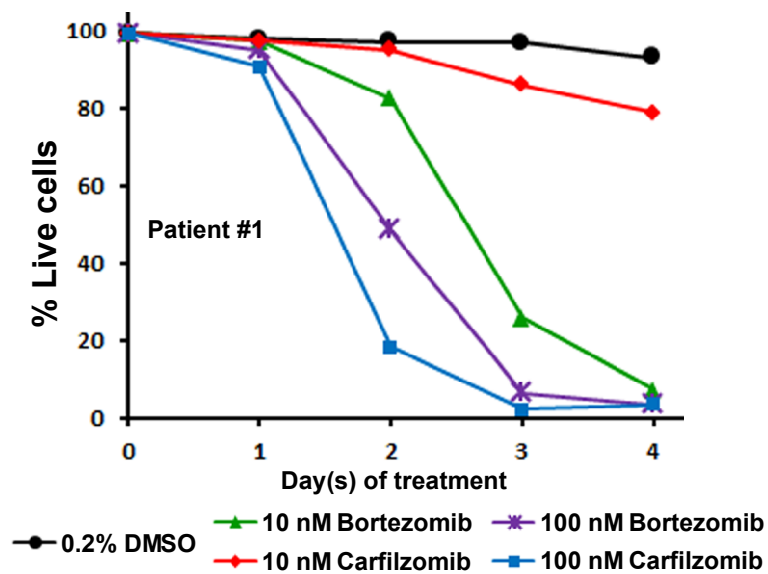**B**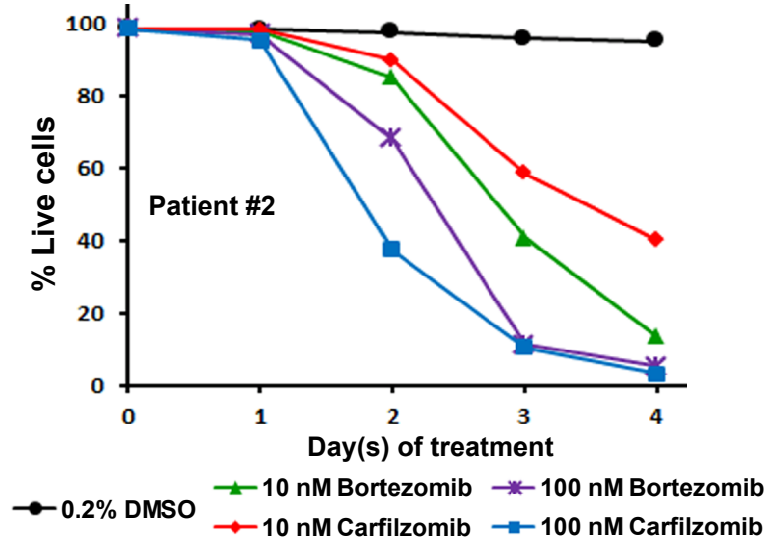**C**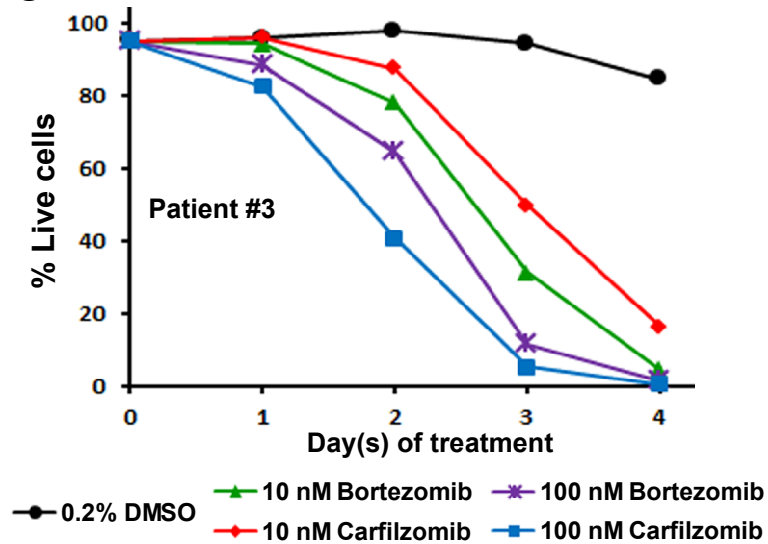

Supplement: S7 Fig — A., B., & C. Primary CD4+ T cells isolated from ART-suppressed HIV-1-infected patient #1 (A), #2 (B) and #3 (C) were treated with the indicated drugs for 4 days. An aliquot of cells from each treatment was collected on the indicated days, stained with LIVE/DEAD Cell Stain Kit (Invitrogen, L34955), and subjected to flow cytometry to quantify the percentages of live cells. (PDF) [file ppat.1007498.s007.pdf]

**A**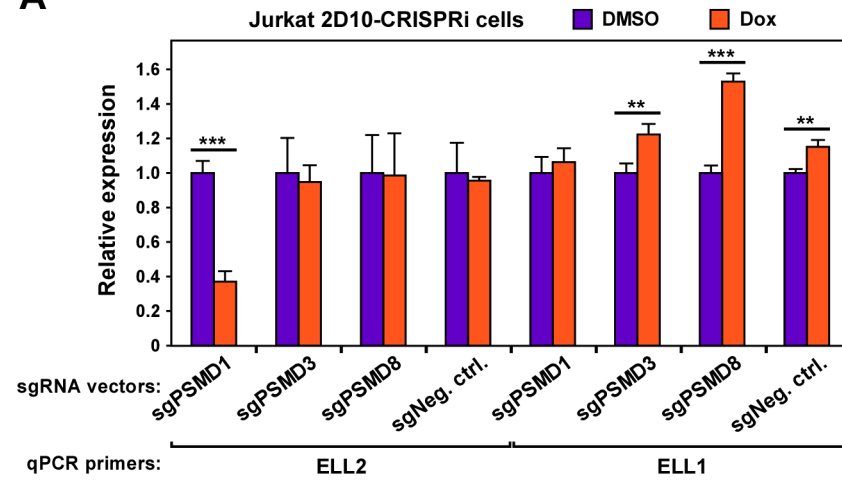**B**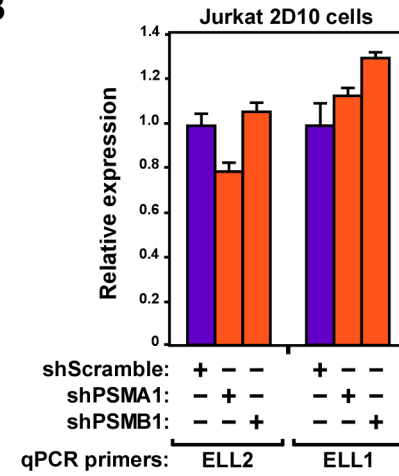

Supplement: S8 Fig — A. & B. Results of RT-qPCR analyses of the mRNA levels of ELL1 and ELL2 in aliquots of the cells treated and examined in Fig 5B & 5C. For each group, the mRNA level in the DMSO-treated cells was set to 1. Error bars represent mean +/- SD from three experimental replicates. Asterisks denote levels of statistical significance calculated by two-tailed Student’s t-test (*: p<0.05, **: p<0.01, and ***: p<0.001). (PDF) [file ppat.1007498.s008.pdf]
