# Supplementary material for: Reiterative Enrichment and Authentication of CRISPRi Targets (REACT) identifies the proteasome as a key contributor to HIV-1 latency
Source: PLoS Pathog. 2019 Jan 15;15(1):e1007498. doi: 10.1371/journal.ppat.1007498 (PMC6333332; doi:10.1371/journal.ppat.1007498)
Supplement: S1 Table — (PDF) [file ppat.1007498.s009.pdf]

Supplemental Table 1. Characteristics of HIV-1–infected study participants

Patient ID	Age (years)	Gender	Ethnicity	Duration of infection (years)	ART regimen	Time on ART (years)	Time on suppressive ART (years)	CD4 count (cells/mm^3)	CD8 count (cells/mm^3)	Viral load (copies/ml)	Peak viral load (copies/ml)	CD4_Nadir (cells/mm3)
1	72	Male	White	23	TCV, FTC/TAF	12	10	725	1024	<40	28699	223
2	62	Male	Hispanic/Latino	25	ABC/TCV/3TC	16.4	10.9	606	407	<40	119870	4
3	58	Male	Black/African American	37	ABC/TCV/3TC	18.4	16.6	235	729	<40	unknown	185
4	50	Male	White	17	TCV/RPV	13	12	380	304	<40	495912	129
5	66	Male	White	34	TCV, FTC/TAF	19	19	489	619	<40	unknown	40
6	67	Male	White	18	ABC/TCV/3TC	12	11	522	544	<40	376658	378
7	57	Male	White	31	RTV, DRV, ABC/TCV/3TC	20	11	615	804	<40	171000	147
8	57	Male	Black/African American	21	EFV/TDF/FTC	10	9	466	555	<40	150000	211
9	60	Male	Black/African American	13	EFV/TDF/FTC	11	10	687	237	<40	312938	153
10	34	Male	White	7	EVG/TDF/FTC/COBI	7	7	293	642	<40	694195	50
11	49	Male	Black/African American	13	FTC/TDF, RTV, DRV	8	8	639	921	<40	286924	208
12	47	Male	Hispanic/Latino	25	ABC/TCV/3TC, ATV	14	10	351	466	<40	357000	56
13	70	Male	White	33	DRV, RTV, TCV, 3TC	24	7	836	889	<40	80410	98

3TC, lamivudine (Epivir); ABC, abacavir (Ziagen); ATV, atazanavir (Reyataz); COBI, cobicistat; DRV, darunavir (Prezista); EFV, efavirenz (Sustiva, Stocrin); EVG, elvitegravir; FTC, emtricitabine (Emtriva); RPV, rilpivirine (Edurant); RTV, ritonavir (Norvir); TAF, tenofovir alafenamide; TCV, dolutegravir (Tivicay); TDF, tenofovir (Viread).
