# Supplementary material for: Reiterative Enrichment and Authentication of CRISPRi Targets (REACT) identifies the proteasome as a key contributor to HIV-1 latency
Source: PLoS Pathog. 2019 Jan 15;15(1):e1007498. doi: 10.1371/journal.ppat.1007498 (PMC6333332; doi:10.1371/journal.ppat.1007498)
Supplement: S2 Table — (PDF) [file ppat.1007498.s010.pdf]

Supplemental Table 2. List of DNA oligonucleotide primers used in this study

Primer	Sequence (5' → 3')
For deep-sequencing:	
CRISPRi_TSS_12_P5	AATGATACGGCGACCACCGAGATCTACACTTGTAGGAAGAGCACACGTCTGAACTCCAGTCACGCACAAAAGGAAACTCACCCT
CRISPRi_TSS_12_P7	CAAGCAGAAGACGGCATACGAGATCTTGTAGTGACTGGAGTTCAGACGTGTGCTCTTCCGATCACGACTCGGTGCCACTTTTTC
CRISPRi_TSS_6_P5	AATGATACGGCGACCACCGAGATCTACAGCCAATGGAAGAGCACACGTCTGAACTCCAGTCACGCACAAAAGGAAACTCACCCT
CRISPRi_TSS_6_P7	CAAGCAGAAGACGGCATACGAGATGCCAATGTGACTGGAGTTCAGACGTGTGCTCTTCCGATCACGACTCGGTGCCACTTTTTC
CRISPRi TSS_seq V2	GTGTGTTTTGAGACTATAAGTATCCCTTGGAGAACCACCTTGTTGG
For qPCR:	
qActB-F	AGAGCTACGAGCTGCCTGAC
qActB-R	AGCACTGTGTTGGCGTACAG
qGAPDH-F	AATCCCATCACCATCTTCCAG
qGAPDH-R	AAATGAGCCCCAGCCTTC
qEnv-F	GAGACAGAGACAGATCCATTCG
qEnv-R	CCAGAAGTTCCACAATCCTCG
qGFP-F	CAGTGCTTCAGCCGCTACCC
qGFP-R	AGTTCACCTTGATGCCGTTCTT
qPSMD1-F	CTGAGCTGACAGATACACTGC
qPSMD1-R	GTGCAAATTCCTTAAGCTGTGG
qPSMD3-F	CGCCTCAACCACTATGTTCTG
qPSMD3-R	AATCAGCCTCTGTGTCCATG
qPSMD8-F	GAACCGTAAAAGCCCCAATC
qPSMD8-R	CCCGATCTCCAGTATGTCAC
qGON4L-F	GAAGTCAAGGAAGAAGGAGGG
qGON4L-R	AGGAGAGGCACAAACATCTG
qCYLD-F	TGGGATGGAAGATTTGATGGAG

qCYLD-R	CATAAAGGCAAGTTTGGGAGG
qNFKBIA-F	GTCTACACTTAGCCTCTATCCATG
qNFKBIA-R	AGGTCAGGATTTTGCAGGTC
qPSMA1-F	GGTTGCATTGAAAAGGGCG
qPSMA1-R	ATCCAAACACTCCTGACGC
qPSMB1-F	GCTGCAATGCTGTCTACAATC
qPSMB1-R	TCTCTGGTAAGACCCTACTGG
qELL1-F	CCTTCTACCTCTCCAACATCG
qELL1-R	ACCGTGATCTTGTCCTGTATG
qELL2-F	GAGACTTACCAGAGCCACAAG
qELL2-R	TTGTCTTTGCCCACATTTGAC
