# Supplementary material for: Reiterative Enrichment and Authentication of CRISPRi Targets (REACT) identifies the proteasome as a key contributor to HIV-1 latency
Source: PLoS Pathog. 2019 Jan 15;15(1):e1007498. doi: 10.1371/journal.ppat.1007498 (PMC6333332; doi:10.1371/journal.ppat.1007498)
Supplement: S3 Table — (PDF) [file ppat.1007498.s011.pdf]

Supplemental Table 3. List of antibodies used in this study

Antibody	Source
rabbit anti-human ELL2	Bethyl A302–505A
rabbit anti-human ELL1	Bethyl A301–645A
rabbit anti-human ENL	Bethyl A302-268A
mouse anti-human AFF4	Abcam ab57077
rabbit anti-human AFF1	Bethyl A302–344A
rabbit anti-human CycT1	Santa Cruz sc-10750
rabbit anti-human AF9	Bethyl A300-595A
rabbit anti-human LARP7	(He et al., 2008)
mouse anti-human IκBα	Santa Cruz sc-1643
rabbit anti-human PSMD1	Bethyl A303-852A
rabbit anti-human PSMD3	Bethyl A303-826A
mouse anti-human PSMD8	Santa Cruz sc-514053
rabbit anti-human PSMA1	Bethyl A303-845A
rabbit anti-human PSMB1	Abcam ab135830
rabbit anti-human Brd4	(Yang et al., 2005)
rabbit anti-human CDK9	(He et al., 2010)
goat anti-mouse-680 nm	Invitrogen A-21057
goat anti-rabbit-680 nm	Invitrogen A-21076

References

He, N., Jahchan, N.S., Hong, E., Li, Q., Bayfield, M.A., Maraia, R.J., Luo, K., and Zhou, Q. (2008). A La-related protein modulates 7SK snRNP integrity to suppress P-TEFb-dependent transcriptional elongation and tumorigenesis. Mol Cell *29*, 588-599.

He, N., Liu, M., Hsu, J., Xue, Y., Chou, S., Burlingame, A., Krogan, N.J., Alber, T., and Zhou, Q. (2010). HIV-1 Tat and host AFF4 recruit two transcription elongation factors into a bifunctional complex for coordinated activation of HIV-1 transcription. Mol Cell *38*, 428-438.

Yang, Z., Yik, J.H., Chen, R., He, N., Jang, M.K., Ozato, K., and Zhou, Q. (2005). Recruitment of P-TEFb for stimulation of transcriptional elongation by the bromodomain protein Brd4. Mol Cell *19*, 535-545.
